# Assessment of antimicrobial activity of [1,2,4]triazolo[4, 3-a]quinoxaline derivatives individually and in combination with levofloxacin

**DOI:** 10.1038/s41598-026-39141-y

**Published:** 2026-02-19

**Authors:** Nasrin Saberi Harooni, Mohammad Reza Naimi-Jamal, Mohammad Ghorban Dekamin, Azar Tahghighi

**Affiliations:** 1 Medicinal Chemistry Laboratory, Clinical Research Department, Pasteur Institute of Iran, Tehran, Iran; 2 Research Laboratory of Green Organic Synthesis and Polymers, Department of Chemistry, Iran University of Science and Technology, Tehran, Iran; 3 Pharmaceutical and Heterocyclic Compounds Research Laboratory, Department of Chemistry, Iran University of Science and Technology, Tehran, Iran

**Keywords:** Triazoloquinoxaline, Antimicrobial agents, Synergistic effect, FE-SEM, Toxicity, Biochemistry, Chemistry, Drug discovery, Microbiology

## Abstract

The need to develop new and effective antimicrobial compounds has become a fundamental requirement of the therapeutic system worldwide. In this study, a series of synthetic 4-amino triazoloquinoxaline derivatives were designed and synthesized. The chemical structure of these compounds was confirmed using spectroscopic methods (^1^H-NMR, ^13^C-NMR, FT-IR, and MS). Their antimicrobial activity against several Gram-positive and Gram-negative bacteria was then evaluated. The results of biological experiments showed that the synthetic compounds **5b**, **5d**, and **5h** derivatives have moderate antimicrobial activity against *Staphylococcus aureus*, *Staphylococcus epidermidis*, *Escherichia coli*, *Pseudomonas aeruginosa*, and *Candida albicans* compared to some standard drugs. Non-toxic compounds were selected based on MTT and hemolysis assays, and their synergistic effect in combination with levofloxacin (LEV) was investigated. The findings confirmed the effectiveness of combination therapy in reducing the dose of LEV, compounds **5d** and **5h** against S. *aureus* and *S. epidermidis*, as well as **5d** and **5b** against resistant isolates of MRSA and *P. aeruginosa*, respectively, with a fractional inhibitory concentration index ≤ 0.5. Field-emission scanning electron microscopy confirmed the complete damage of *S. aureus*, *S. epidermidis*, and *P. aeruginosa* following exposure to the combination therapy of compounds **5b**, **5d**, and **5h** with LEV. This new synergistic therapy has the potential to be used as a novel approach of treating infectious diseases after future investigations.

**Supplementary Information:**

The online version contains supplementary material available at 10.1038/s41598-026-39141-y.

## Introduction

**Fig. 1:**
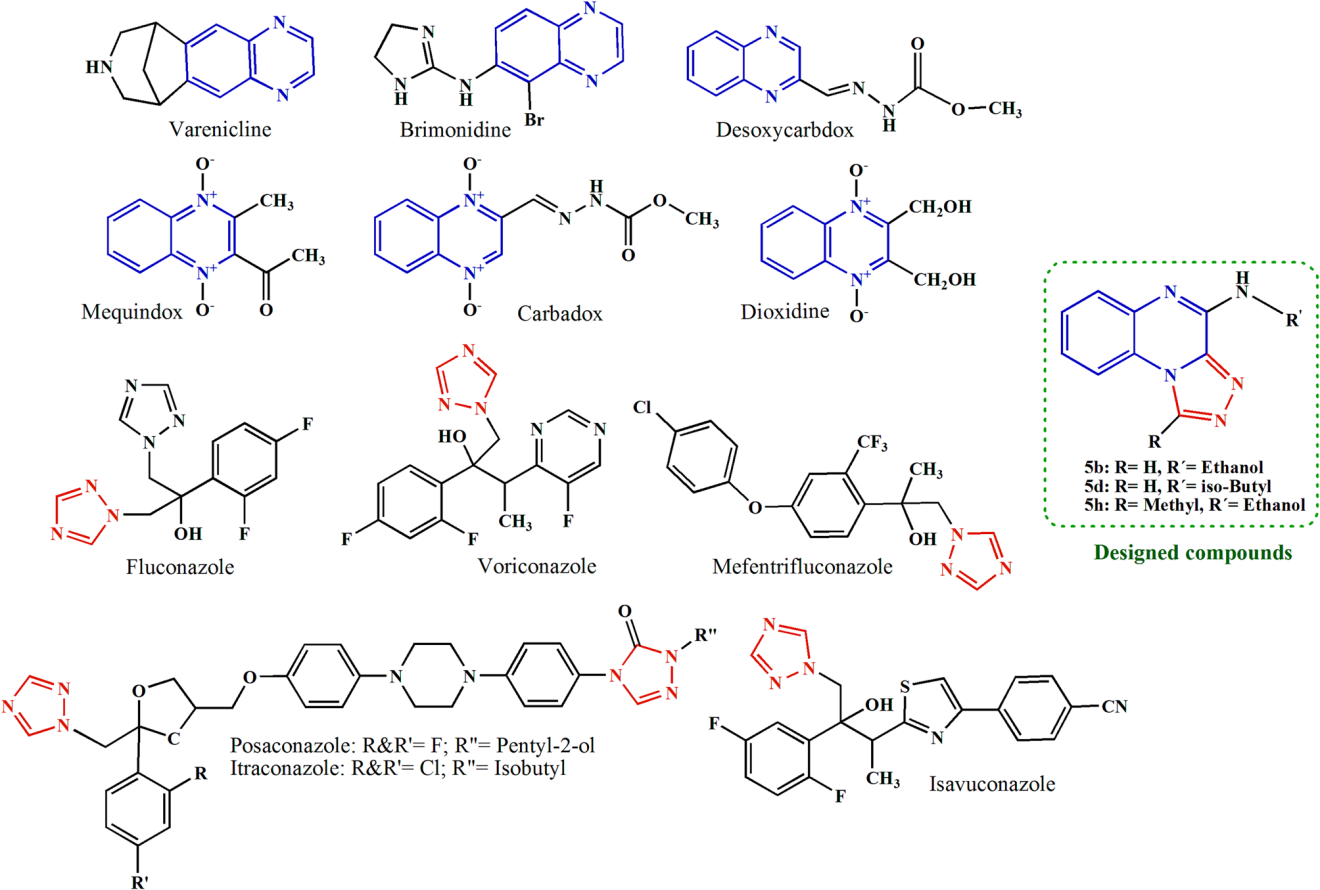
Designed compounds and marketed antimicrobial drugs containing quinoxaline and triazole ring.

Combating microbial infections, particularly with the emergence and development of drug-resistant strains, is a critical global health issue in the present century^1^. The development of new chemical antimicrobial agents can be a suitable response to this global challenge. Heterocyclic compounds have always been of interest to scientists for designing and synthesizing new drugs due to their diverse structures, ease of structural modification , remarkable chemical adaptability, and wide range of biological activities, including antimicrobial activity^2–7^. Heterocyclic compounds play a pivotal role in pharmaceutical chemistry and drug discovery due to their remarkable structural diversity and distinctive chemical reactivity. The incorporation of heteroatoms such as nitrogen, oxygen, or sulfur into cyclic frameworks profoundly influences electronic distribution, allowing fine-tuning of essential physicochemical properties, including lipophilicity, polarity, and metabolic stability. Moreover, the presence of heteroatoms enables diverse intermolecular interactions with biological targets through hydrogen bonding and electrostatic interactions, thereby enhancing binding affinity and overall biological activity. Among the known heterocyclic structures, compounds containing triazoloquinoxaline rings are of particular importance due to their broad spectrum of biological activities^8–10^. The triazoloquinoxaline scaffold, a fused tricyclic ring consisting of a bicyclic quinoxaline ring fused with a triazole ring, is a well-known heterocyclic structure with suitable chemical, physical, and biological properties, which have earned a special place in recent years for designing and synthesizing new drugs^11–13^. In the quinoxaline ring, the incorporation of benzene and pyrazine rings donates unique structural, electronic, physicochemical, and reactive properties to this bicyclic ring^14^. The powerful electron-withdrawing properties of quinoxalines, related to the nitrogen atoms in the pyrazine ring and the selection of suitable substitutions at the 2- and 3-positions of the quinoxaline ring, which are the most flexible and best positions for chemical modifications, influence its electronic properties, providing fine adjustments of binding affinities to specific biological targets. Hydrophilic/hydrophobic substituents in these positions, as well as the nitrogen atoms of the pyrazine ring, support effective interactions including π-stacking, hydrogen bonding, acid-base, and other polar/non-polar interactions with specific macromolecular targets. Additionally, modification of hydrophilicity/hydrophobicity of quinoxaline derivatives by suitable substitutions at specific positions can enhance cellular uptake and bioavailability by improving their physicochemical properties^15^. Quinoxalines exhibit notable thermal stability and suitable solubility characteristics in organic solvents. Their electron-rich aromatic system contributes to redox behavior and leads to their involvement in modifying oxidative stress pathways, a key mechanism in the treatment of infectious diseases and cancer^16^. These properties make quinoxaline an impressive scaffold in the drug discovery field. Several drugs in the market, including dioxidine, mequindox, carbadox, and desoxycarbadox, which incorporate the quinoxaline ring, are used as antibacterial agents (Fig. 1).

1,2,4-Triazole, a five-membered heterocyclic ring consisting of two carbon and three nitrogen atoms, is another important heterocyclic scaffolds in pharmaceutical chemistry^17,18^. All the atoms are sp^2^-hybridized, and the delocalization of six π electrons around the ring confers aromatic character and planar structure^18,19^. Chemically, 1*H*-1,2,4-triazole undergoes both electrophilic and nucleophilic substitution reactions. Due to its high electron density, electrophilic substitution occurs at the nitrogen atoms only. Since carbon atoms are attached to two electronegative nitrogen atoms and become π-deficient, they are susceptible to nucleophilic attack. These structural characteristics lead to specific features such as weak basicity, various dipole moments, polarity, rigidity, and the ability to act as dual hydrogen bond donor/acceptor in triazole rings^20^.

These properties make triazoles a significant platform in medicinal chemistry and chemical biology, giving them key roles in various biological mechanisms related to infections, inflammation, cancer, neurodegeneration, and oxidative stress^21,22^. Triazoles like fluconazole, itraconazole, voriconazole, isavuconazole, and posaconazole, which are marketed pharmaceuticals, i all contain a 1,2,4-triazole nucleus and are employed in the treatment of *Candida* infections (Fig. 1). Recently, mefentrifluconazole was introduced to the European market as an effective fungicide^23^. Some research has confirmed antimicrobial activity of bioactive molecules with a 1,2,4-triazole core^24^.

The combination of these two attractive structures in the form of a fused molecule has led to the production of a new structure called triazoloquinoxaline, which is expected to have outstanding biological activities, considering the advantages of both skeletons^8^. Numerous studies have shown that the combination of two or more bioactive structures in one molecule can lead to increased efficiency and improved pharmacokinetic and pharmacodynamic properties. For example, studies by El Faydy et al. (2024) and Al-Marhabi et al. (2015) have shown that the hybridization of triazole fused with quinoline or quinoxaline has stronger antimicrobial activity than the individual compounds^25,26^. Previous studies have also shown that triazoloquinoxaline derivatives can play an effective inhibitory role by targeting enzymes and biological pathways in bacteria and fungi^27^. These structures, due to their electrophilic nature and ability to interact with biologically active sites, have great potential for the design and discovery of novel antimicrobial agents.

It should be noted that the global crisis of antimicrobial drug resistance has driven researchers to develop innovative strategies for combating drug-resistant infections. One of these promising approaches involves exploiting synergistic effects between novel synthetic compounds and conventional antimicrobial agents. Synergism is the state in which the combination of two or more therapeutic agents produces an effect that is stronger than the sum of their individual effects. The mode of action in combination therapy mainly differs from that of the same drugs acting individually. This phenomenon can diminish the dosage of conventional drugs, reduce side effects, and increase the effectiveness of treatment^28,29^. In recent years, synthetic heterocyclic compounds, especially those containing skeletons such as triazole^30,31^, quinoxaline^12^, pyrimidine^3^, nitrothiophene^7^, chromone-isoxazoline^2^, thiadiazole^32^, dioxoisoindolin^33^, and flavonoids^34^ have received much attention due to their potent antimicrobial activity and potential for synergistic effects with known drugs.

Studies have shown that some of the synthetic heterocyclic compounds can enhance the performance of antibiotics such as tobramycin, rifampicin, metronidazole, clarithromycin, novobiocin, chloramphenicol, colistin, aminoglycosides, and β-lactam antibiotics, and even be effective against bacteria resistant to these drugs^33–37^. In this regard, the investigation and identification of compounds capable of synergistic interactions with conventional drugs can pave the way for the development of new and effective combination therapies.

In this study, with the aim of identifying new and effective antibacterial compounds, a series of triazoloquinoxaline derivatives were designed and synthesized, and the antimicrobial activity of selected compounds and their synergistic effect in combination with LEV were evaluated. FE-SEM was applied to evaluate morphological alterations in bacterial strains exposed to synergistically antibacterial compounds. The cytotoxicity of the synthetic compounds against the L929 cell line and their hemolytic activity on erythrocytes were also assessed. The ultimate goal is to identify compounds with potential for use in enhanced combination therapies and to address the growing challenge of antimicrobial resistance.

## Results

### Chemistry

**Fig. 2:**
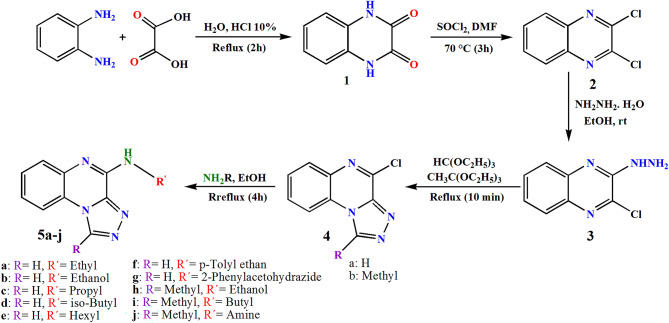
Synthetic route for intermediates and final compounds **5a-j**.

This present study focused on synthesis of a novel of [1,2,4]triazolo[4,3-a]quinoxaline derivatives containing alkyl/aryl amine moieties (**5a-j**). This was successfully achieved via a five-step synthetic approach, and the compounds were subsequently investigated for their antimicrobial activities, both individually and in combination with LEV. The synthetic route commenced with cyclization of benzene diamine in the presence of oxalic acid and hydrochloric acid in water^38^. In the next step, quinoxaline-2,3(1*H*,4*H*)-dione (**1**) was reacted with thionyl chloride to substitute the oxygen atoms with chlorine atoms^39^. Subsequently, 1 equivalent of 2,3-dichloroquinoxaline (**2**) underwent an SN_2_ reaction with 1.2 equivalent of hydrazine hydrate in ethanol^40^. In the fourth step, refluxing 2-chloro-3-hydrazineylquinoxaline (**3**) with triethyl orthoformate or triethyl orthoacetate afforded 4-chloro-[1,2,4]triazolo[4,3-a]quinoxaline and 4-chloro-1-methyl-[1,2,4]triazolo[4,3-a]quinoxaline (**4a-b**) in high yields via an intramolecular cyclization cascade^41^. Finally, C-N bond formation of the triazoloquinoxaline core with different primary amines was done in ethanol under reflux condition. Ten compounds, [1,2,4]triazolo[4,3-a]quinoxaline (**5a-g)** and 1-methyl-[1,2,4]triazolo[4,3-a]quinoxaline (**5i-j**) bearing alkyl/benzyl amine substituents, were synthesized through nucleophilic substitution of intermediates **4a-b** with various electron-donating amines in high yields (Fig. 2).

The structural elucidation of the synthesized triazoloquinoxaline derivatives (**5a–j**) was achieved through a combination of melting point analysis and comprehensive spectroscopic techniques, including FT-IR, ^1^H-NMR, ^13^C-NMR, and mass spectrometry. Due to the close structural similarity among the final compounds, comparable spectral features were observed across the series, particularly with respect to their shared functional groups. In the FT-IR spectra, all compounds exhibited characteristic aromatic C–H (sp²) stretching vibrations in the range of 3194–3025 cm⁻¹ and aliphatic C–H (sp³) stretching bands at 2945–2886 cm⁻¹. The presence of the NH moiety in the triazoloquinoxaline framework was confirmed by distinct absorption bands at 3341–3303 cm⁻¹, which correlated well with the singlet signals observed in the^ 1^H-NMR spectra at 7.95–8.29 ppm. These findings are consistent with reported FT-IR and^ 1^H-NMR data for related triazoloquinoxaline systems described in the literature^38–41^. The aromatic C = C stretching vibrations appeared prominently at 1676–1589 and 1484–1450 cm⁻¹, confirming the presence of the quinoxaline aromatic core. These FT-IR features were further supported by ^1^H-NMR aromatic proton signals in the range of 7.11–8.22 ppm and^ 13^C-NMR resonances between 116 and 146 ppm, corresponding to the aromatic carbons of the quinoxaline ring.

The formation of the triazole ring was conclusively evidenced by the appearance of characteristic C = N stretching bands in the FT-IR spectra at 1589–1522 cm⁻¹. These bands correlated well with^ 13^C-NMR signals at 138–146 ppm, attributable to the triazole ring carbons. In addition, the^ 1^H-NMR spectra displayed singlet signals corresponding to the triazole CH proton at 9.97–10.24 ppm and the triazole methyl (CH₃) group at 3.04–3.16 ppm. These proton resonances were further supported by ^13^C-NMR signals at approximately 139 and 146 ppm for triazole carbons and at 14–32 ppm for aliphatic sp³-hybridized methyl carbons, respectively. Notably, compounds **5b** and **5h** exhibited additional broad FT-IR absorption bands at 3463–3443 cm⁻¹, indicative of hydroxyl (OH) functionalities. The presence of these hydroxyl groups was further corroborated by corresponding downfield signals in the^ 1^H-NMR spectra, confirming successful functionalization. Detailed^ 13^C-NMR analysis further substantiated the proposed structures, with characteristic signals assigned to the quinoxaline ring carbons at 116 ppm (C_5_), 122 ppm (C_4a_), 123 ppm (C_6_), 126 ppm (C_8_), 127 ppm (C_7_), 137 ppm (C2), 138 ppm (C8a), and 149 ppm (C_1_). These assignments are fully consistent with the^ 1^H-NMR data and confirm the integrity of the fused triazoloquinoxaline scaffold. Finally, mass spectrometric analysis provided confirmation of the molecular structures. All synthesized compounds exhibited prominent molecular ion peaks corresponding to (M^+^), which matched the calculated molecular weights of the target compounds. The observed mass fragmentation patterns and molecular ion peaks are in excellent agreement with those reported for structurally related triazoloquinoxaline derivatives, further validating the successful synthesis. Overall, the combined FT-IR, ^1^H-NMR, ^13^C-NMR, and MS data unequivocally confirm the proposed structures of the synthesized triazoloquinoxaline derivatives, and the spectral characteristics are in strong agreement with previously published studies on analogous heterocyclic compounds.

### Antimicrobial activity

The antibacterial activity of all synthetic compounds with diameters of inhibition zones (DIZ) ≥ 12 mm was evaluated under laboratory conditions using the microdilution method against various susceptible bacterial strains, including *S. aureus* and *S. epidermidis* as Gram-positive bacteria and *E. coli*, and *Pseudomonas aeruginosa* as Gram-negative one. The results of the antibacterial activity of the aforementioned compounds against all tested strains are shown in Table 1. Compounds **5b**, **5d**, and **5 h** presented the best antimicrobial activity against several microorganisms compared with others derivatives.

**Table 1:** Antimicrobial activity of synthetic triazoloquinoxaline compounds (MIC, MBC, and MFC; mg/mL).

Sample	*S. aureus* (ATCC 29213)		*S. epidermidis* (ATCC 12228)		*P*. aeruginosa (ATCC 27853)		*E. coli* (ATCC 25922)		*C. albicans* (ATCC 10231)	
	MIC	MBC	MIC	MBC	MIC	MBC	MIC	MBC	MIC	MFC
**5a**	0.125	0.125	0.25	0.25	0.125	0.125	0.125	0.125	0.125	0.125
**5b**	0.0937	0.125	0.0625	0.125	0.0625	0.125	0.0937	0.125	0.0625	0.125
**5c**	0.25	0.25	0.25	0.25	0.125	0.125	0.125	0.25	0.25	0.25
**5d**	0.0625	0.125	0.0625	0.0625	0.0352	0.125	0.0625	0.125	0.0625	0.125
**5e**	> 0.25	> 0.25	> 0.25	> 0.25	> 0.25	> 0.25	> 0.25	> 0.25	0.125	0.125
**5f**	0.125	0.125	0.25	0.25	0.125	0.125	0.125	0.125	0.125	0.125
**5g**	0.125	0.125	0.1875	0.25	0.125	0.125	0.125	0.125	0.125	0.125
**5h**	0.0625	0.125	0.125	0.125	0.0625	0.125	0.125	0.125	0.0625	0.125
**5i**	0.125	0.125	0.125	0.25	0.125	0.125	0.125	0.125	0.125	0.125
**5j**	0.125	0.125	0.25	0.25	0.125	0.125	0.0937	0.125	0.125	0.125
**Lev**	0.00025	0.0005	0.00012	0.00025	0.00025	0.0005	0.002	0.0005	-	-
**Amk**	0.005	0.005	0.005	0.005	0.003	0.006	0.001	0.001	-	-
**Pen**	0.0015	0.003	0.0015	0.003	**-**	**-**	-	-	-	-
**Flu**	-	-	-	-	-	-	-	-	0.002	0.005

MIC: Minimum inhibitory concentration; MBC: Minimum bactericidal concentration; MFC: Minimum fungicidal concentration;* S. aureus*: *Staphylococcus aureus*; *S. epidermidis*: *Staphylococcus epidermidis*; *E. coli*: *Escherichia coli*; *P. aeruginosa*: *Pseudomonas aeruginosa*; *C. albicans*: *Candidia albicans*; Lev: Levofloxacin (4 µg/mL); Pen: Penicillin (12 µg/mL); Amk: Amikacin (10 µg/mL); Flu: Fluconazole (150 µg/mL). DMSO had inhibitory activity at 12.5%v/v.

One of the microorganism screened was *S. aureus*, a Gram-positive, spherically shaped bacterium that has a higher occurrence among hospital workers and patients^42^. The MIC of compounds **5b**, **5d**, and **5h** against *S. aureus* ranged from 0.0625 to 0.0937 mg/mL. The second bacterial strain used herein was *S. epidermidis*, a Gram-positive, cocci-shaped bacterium that forms part of the normal microflora of the skin. However, under some conditions, most importantly, the implantation of a medical device, *S. epidermidis* can switch from a colonizing to an invasive lifestyle^43^. Compounds **5b** and **5d** against *S. epidermidis* displayed MIC values of 0.0625 mg/mL. *P. aeruginosa*, a Gram-negative, rod-shaped bacterium, was another bacterial strain tested, with MIC values of 0.0625, 0.0352, and 0.0625 mg/mL for compounds **5b**, **5d**, and **5h**, respectively. It is a significant opportunistic pathogen, causing various infections in humans, particularly in those with weakened immune systems^44^. The final bacterial strain used herein was *E. coli*, a rod-shaped coliform bacterium and Gram-negative bacillus that is a causative organism of many diarrheal illnesses, including traveler’s diarrhea and dysentery. Compounds **5b** (MIC = 0.0937 mg/mL) and **5d** (MIC = 0.0625 mg/mL) were active against *E. coli*. Nonetheless, screening of compound **5h** against *S. epidermidis* and *E. coli* showed the least potential of bioactivity compared with **5b** and **5d**. *C. albicans*, the sole fungus examined in this study, is a common commensal organism that colonizes the oropharyngeal cavity, gastrointestinal tract, healthy skin, and genital tract. Various factors can disrupt the normal homeostasis of Candida, leading to its transition from a harmless member of the normal flora to a pathogenic and opportunistic infectious agent^45^. All compounds **5b**, **5d**, and **5h** showed similar MIC values of 0.0625 mg/mL against *C. albicans*. Although these values were higher than those of the reference drugs (MIC ≤ 0.0015 mg/mL) (Table 1).

**Fig. 3:**
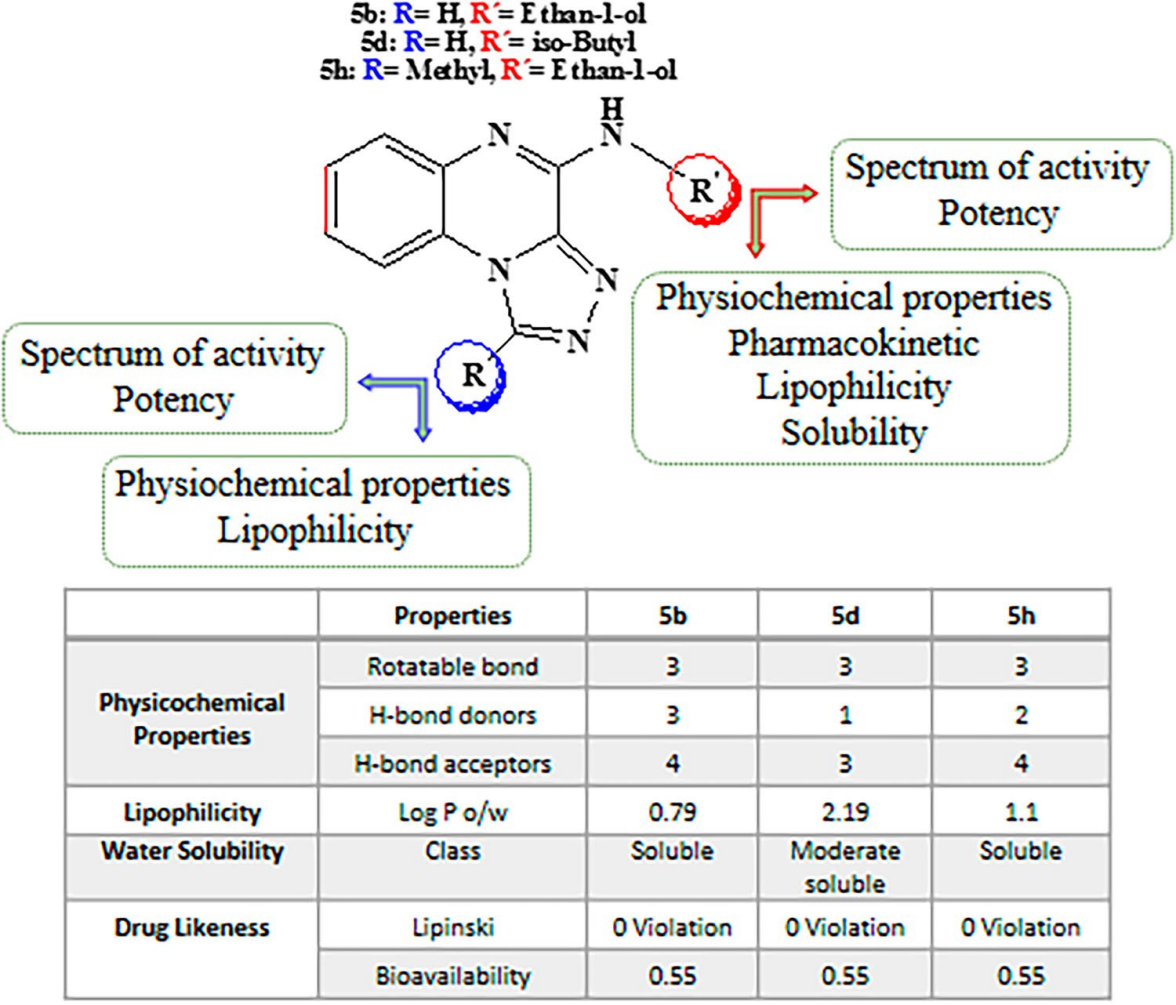
Structure-activity relationship and in silico prediction of properties of compounds **5b**, **5d**, and **5h**.

Small modification of the moieties around the trizoloquinoxaline core significantly influenced the antimicrobial activity and physicochemical profile of the derivatives (Fig. 3; Table 1). Compound **5d,** with a branched iso-butyl amine substitution on the quinoxaline moiety and without any substitution on the triazole moiety showed broad spectrum activity with lower MICs. This can be related to the higher lipophilicity of this compound compared with compounds **5b** and **5h** (Fig. 3). Compound **5e** with a linear hexyl substitution, showed no activity against bacterial strains. Comparison between compounds **5b** and **5h,** with similar ethanolamine substitution on the quinoxaline ring but different substitution on the triazole ring, showed a minor reduction in spectrum activity and potency of compound **5h** with methyl substitution, that may be related to steric hindrance around the triazoloquinoxaline ring. The physicochemical characteristics of compounds **5b**, **5d**, and **5h** were predicted by SwissADME web tool, which is freely available at http://www.swissadme.ch, and confirmed drug-likeness and medicinal chemistry friendliness of these compounds (Fig. 3).

### Synergistic effects

**Fig. 4:**
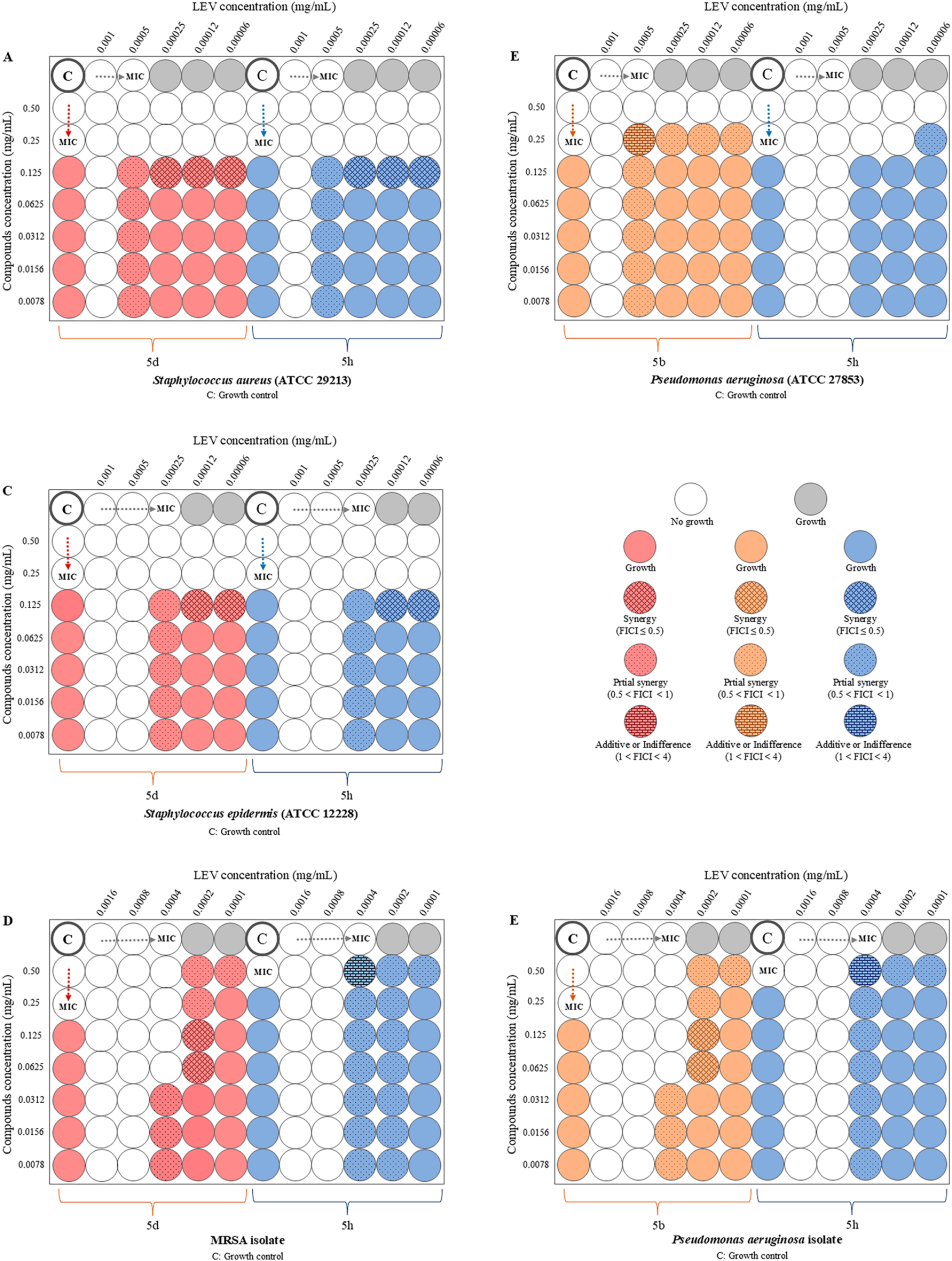
Synergistic effect of synthetic compounds in combination with levofloxacin against sensitive bacterial strains and clinical isolates. MIC: Minimum inhibitory concentration.

Figure 4 shows the antibacterial effects of compounds **5b**, **5d**, and **5h**, both individually and in combination with levofloxacin, against sensitive strains of *S. aureus*, *S. epidermidis*,* E. coli*, and *P. aeruginosa,* as well as clinical isolates of methicillin-resistant *S. aureus* (MRSA) and *P. aeruginosa*, as assessed by the checkerboard assay. The results demonstrated a synergistic effect between these compounds and levofloxacin against *S. aureus* (MICs: **5d** & **5h** = 0.25 mg/mL and LEV = 0.0005 mg/mL; synergy concentration of **5d** & **5h**/LEV = 0.0625/0.00003 mg/mL), *P. aeruginosa* (MICs: **5b** & **5h** = 0.25 mg/mL and LEV = 0.0005 mg/mL; partial synergy concentration of **5b** & **5h**/LEV = 0.125/0.00003 mg/mL and **5b**/LEV = 0.0039/0.00025 mg/mL), and *S. epidermidis* (MICs: **5d** & **5 h** = 0.25 mg/mL and LEV = 0.00025 mg/mL; synergy concentration of **5d** & **5h**/LEV = 0.0625/0.00003 mg/mL). Synergistic and partial synergistic effect was also observed for resistant isolates of MRSA (MICs: **5d** = 0.25 mg/mL, **5 h** = 0.5 mg/mL, and LEV = 0.0004 mg/mL; synergy concentration of **5d**/LEV = 0.0312/0.0001 mg/mL and partial synergy concentration of **5 h**/LEV = 0.0039/0.0001 mg/mL), and *P. aeruginosa* (MICs: **5b** = 0.25 mg/mL, **5h** = 0.5 mg/mL, and LEV = 0.0004 mg/mL; synergy concentration of **5b**/LEV = 0.0312/0.0001 mg/mL and partial synergy concentration of **5h**/LEV = 0.0039/0.0002 mg/mL).

### Toxicity assay

**Fig. 5:**
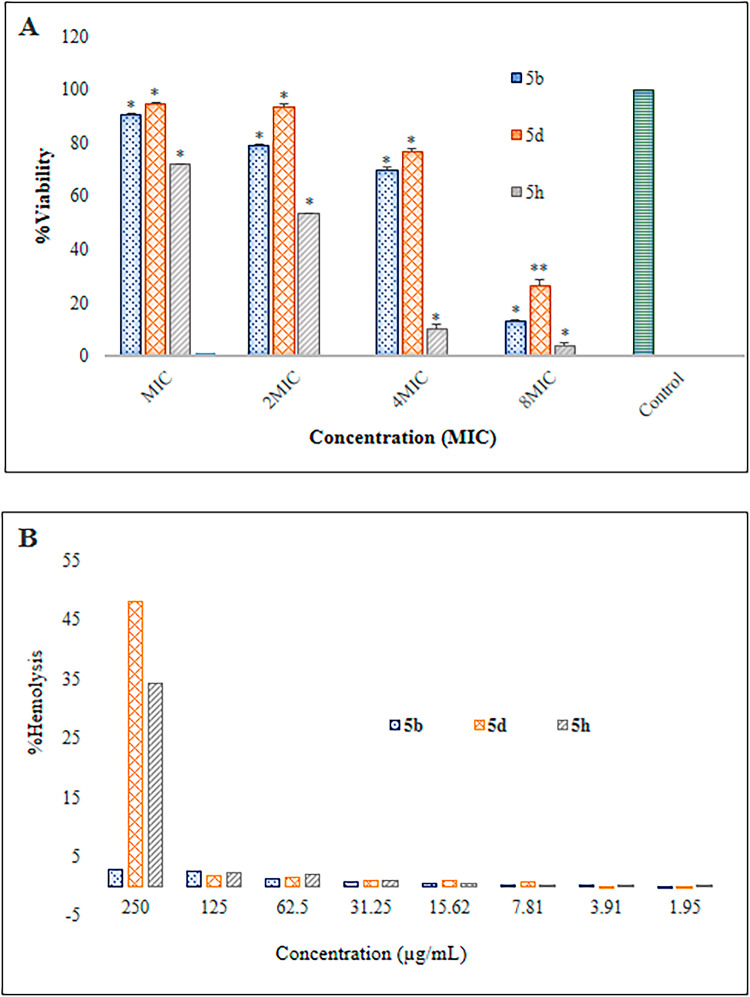
(**A**) Cell viability percentage of L929 (NCBI C161) cells 24 h after incubation with synthetic compounds **5b**, **5d**, and **5h** at various concentrations (MIC, 2MIC, 4MIC, and 8MIC). Data are presented as the mean ±standard deviation from four independent experiments (*n* = 4). In a comparison of all concentrations of compounds (**5b**, **5d**, and **5h**), *p* < 0.05 marked with * indicates different averages of cell viability and a significant relationship between different doses and cell viability. (**B**) The hemolysis percentage at various concentrations of antibacterial compounds (**5b**, **5d**, and **5h**).

The compounds **5b**, **5d**, and **5h** were selected for MTT assay using a mouse fibroblast cell line to assess their safety profiles at concentrations of MIC, 2MIC, 4MIC, and 8MIC (Fig. 5A). Compounds **5b** and **5d** demonstrated a favorable safety profile at MIC concentrations, with cell viability ranging from 90.37% to 94.65%. At 2MIC concentrations, both compounds also showed relative safety, with cell viability ranging from 79.09% to 93.50%. In contrast, compound **5h** exhibited a toxic profile at the MIC level, with 72.12% cell viability. At highest concentration (8MIC), all compounds showed cytotoxicity, with cell viability ≤ 26.42% (Fig. 5A).

The hemocompatibility of compounds **5b**, **5d**, and **5h** with erythrocytes was evaluated at concentrations ranging from 0.002 to 0.25 mg/mL (Fig. 5B). The positive and negative controls resulted in 100% and 11.74% hemolysis, respectively. DMSO, used as the solvent, showed hemolysis comparable to the negative control and was considered safe at the concentration used for compound solubilization. According to the hemolysis assay results, none of the compounds exhibited hemolytic activity at MIC or higher concentrations, with %hemolysis = 2.73% (Fig. 5B). However, compounds **5d** and **5h** showed hemolytic effects at the highest tested concentration (0.25 mg/mL). These findings suggest that the tested compounds are non-toxic to erythrocytes and could be considered promising candidates for further development, pending comprehensive in vivo toxicity and pharmacokinetic studies.

The evaluation of the selectivity index (SI), which indicates the relationship between cytotoxicity against normal cells and antibacterial activity, showed that compounds with an SI value ≥ 0.39 have a favorable safety profile in vitro, suggesting selectivity towards bacterial cells over normal human cells (Table 2). The SI values indicate higher selectivity of compounds toward bacterial cells than normal human cells.

**Table 2:** Selectivity index of synthetic triazoloquinoxaline compounds (CC_50_ and MIC mg/mL).

Sample	*S. aureus* (ATCC 29213)			*S. epidermidis* (ATCC 12228)		*P*. *aeruginosa* (ATCC 27853)		*E. coli* (ATCC 25922)		*C. albicans* (ATCC 10231)	
	CC_50_	MIC	SI	MIC	SI	MIC	SI	MIC	SI	MIC	SI
**5b**	0.2882	0.0937	0.49	0.0625	0.66	0.0625	0.66	0.0937	0.49	0.0625	0.66
**5d**	0.2761	0.0625	0.64	0.0625	0.64	0.0352	0.91	0.0625	0.64	0.0625	0.64
**5h**	0.1532	0.0625	0.39	0.125	0.088	0.0625	0.39	0.125	0.088	0.0625	0.39

CC_50_: 50% Cytotoxic concentration; MIC: Minimum inhibitory concentration; SI: Selectivity index calculated log [CC_50_/MIC]; *S. aureus*: *Staphylococcus aureus*; *S. epidermidis: Staphylococcus epidermidis*; *E. coli*: *Escherichia coli; P. aeruginosa: Pseudomonas aeruginosa*; *C. albicans*: *Candidia albicans*.

### Cellular morphology of synthetic compounds

**Fig. 6:**
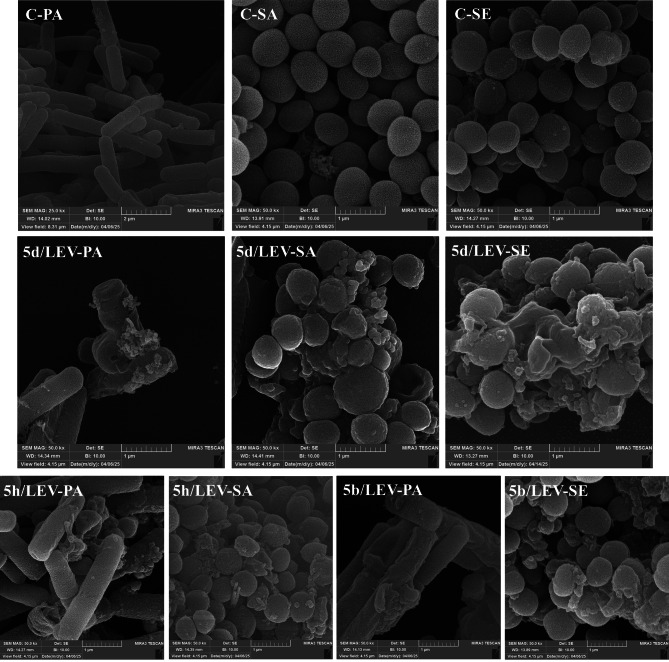
FE-SEM images compare the control with treated sensitive bacterial strains exposed to synergistic concentration of compounds **5b**, **5d**, and **5h** with LEV after 18 h exposure time with 50 kx magnification. PA: *Pseudomonas aeruginosa*; SA: *Staphylococcus aureus*; SE: *Staphylococcus epidermidis*; C: Control; LEV: Levofloxacin.

The antibacterial efficacy of compounds **5b**, **5d**, and **5h** in synergistic concentrations with LEV against *P. aeruginosa*, *S. aureus*, and *S. epidermidis* were determined by evaluating morphological changes of bacterial cells after 18 hours of exposure via FE-SEM analysis (Fig. 6). Untreated cells (controls) exhibited intact cell envelope and smooth surface. Figure 6 shows clear deformation of *P. aeruginosa*, *S. aureus*, and *S. epidermidis* cells treated with a synergistic concentration of **5d**/LEV. Severely deformed, pierced, or indented structures with spilled-out cell contents and, in some cases, indistinct cell boundary walls were observed in treated bacteria (Figs: **5d**/LEV-PA, **5d**/LEV-SA, and **5d**/LEV-SE). When *P. aeruginosa* cells were treated with compound **5b**/LEV and **5h**/LEV at synergistic concentrations (Figs: **5b**/LEV-PA and **5h**/LEV-PA), the cell surface morphology changed, showing membrane distortions, surface indentations, appendages, complete rupture, and release of intracellular components. Similarly, exposure of *S. epidermidis* and *S. aureus* strains with **5b**/LEV and **5h**/LEV, respectively, resulted in dimpled/deformed surfaces, complete rupture, leakage of cellular contents, and distinct appendages on the membrane. According to the FE-SEM images, all bacterial strains were completely destroyed, confirming the effective bactericidal activity of the synergistic concentrations (Fig. 6).

## Discussion

A new series of heterocyclic triazoloquinoxaline derivatives was synthesized via a simple synthesis route, without the need for harsh purification methods and with high yields. The structures of the compounds were confirmed by spectroscopic methods through the identification of distinctive signals, including the CH proton and carbon of the triazole ring, the CH_3_ moiety on the triazole ring, as well as the splitting patterns of quinoxaline ring protons and carbons, and aliphatic substitutions in the ¹H and ¹³C-NMR spectra, the amine absorption band in the FT-IR spectrum, and the molecular ion peak in the mass spectrum. The compounds exhibited moderate antibacterial effects, which can likely be attributed to their chemical structures, enabling interaction with bacterial biological targets but limiting their capacity to exert a strong antibacterial effect independently. These observations are in good agreement with previous studies, which have consistently reported similar antimicrobial activities for several triazoloquinoxaline derivatives bearing diverse substituents at different positions of the heterocyclic ring against a broad spectrum of microbial strains^8,46,47^.

Results of the present study indicated that branched substituent isobutyl and hydrophilic substitution of ethanol improved antibacterial activity in compounds **5b**, **5d**, and **5h**. While, linear alkyls, amine, and aromatic substitutions showed weak antibacterial activity. The better antibacterial activity of triazoloquinoxaline derivatives containing isobutyl and ethanol substitutions can be rationalized by a suitable hydrophilic-lipophilic balance, which may facilitate bacterial membrane penetration and potent interactions with hydrophobic active sites of bacterial targets. Conversely, other substitutions led to reduced membrane penetration and weaker target affinity, likely due to excessive lipophilicity, poor target fitting, ionization at physiological pH, or steric hindrance^48^. Various studies have confirmed the importance of substitutions on heterocyclic compounds in antibacterial activity^6,7,49,50^. Fortunately, compounds **5b**, **5d**, and **5h** were not toxic at MIC concentrations, and compounds **5b** and **5d** were also non-toxic even at twice the MICs. Moreover, none of the tested compounds exhibited hemolytic effect against red blood cells. This represents one of the key strengths of these compounds, which has also been emphasized in other studies ^6,51^.

Unlike some studies that have focused solely on the antibacterial activity of triazoloquinoxaline, another strength of our study is the observation of potential synergistic interactions between LEV and triazoloquinoxaline derivatives. These interactions emphasize enhanced therapeutic efficacy and reduced required concentrations of both the reference drug and the tested synthetic compounds against sensitive strains and resistant isolates. Severe damage to bacterial cells after exposure to synergistic concentrations of the compounds with LEV was confirmed by FE-SEM analysis. The observed synergistic effect may result from several possible mechanisms. First, these compounds may increase the permeability of the bacterial cell wall to LEV, thereby facilitating greater antibiotic uptake and enhancing its efficacy. Second, they may target the defense mechanisms of the pathogenic microorganism, reducing their inherent resistance and allowing LEV to act more effectively. Additionally, it is possible that triazoloquinoxaline derivatives and LEV exert their effects on different macromolecule targets within the microorganism, which can synergistically potentiate their combined antimicrobial activity^52^.

These findings provide valuable insights into the rational design of combination therapeutic strategies for targeting antibiotic-resistant bacteria. Employing triazoloquinoxaline derivatives as enhancers of LEV activity represents a promising approach to overcome bacterial resistance and potentially reduce antibiotic dosages. Notably, this study demonstrates that even compounds with moderate intrinsic antibacterial activity can play a crucial role in enhancing treatment outcomes when used in combination therapies. However, despite these promising results, several limitations should be acknowledged. The antimicrobial and toxicity evaluations were conducted exclusively in vitro using standard strains and a limited number of clinical isolates; therefore, the efficacy of these compounds in complex in vivo infection models remains to be established. Furthermore, while SwissADME predictions offered insights into drug-likeness, these computational estimates require experimental validation through pharmacokinetic and metabolic stability studies. Finally, further investigations are warranted to elucidate the mechanisms underlying the observed synergistic effects. Addressing these aspects in future research will be essential for paving the way toward the development of innovative therapeutic agents.

## Conclusion

This study describes the design and synthesis of a new series of triazoloquinoxaline derivatives and evaluated their antimicrobial and synergistic potential. Spectroscopic analyses confirmed the structures of the synthesized compounds, while antibacterial screening identified **5b**, **5d**, and **5h** as derivatives with moderate antimicrobial activity against both Gram-positive and Gram-negative pathogens. The non-toxicity of these compounds was also confirmed by MTT and hemolysis assays. Importantly, combination studies demonstrated a significant synergistic interaction between these derivatives and levofloxacin , particularly against *S. aureus*, *S. epidermidis*, MRSA, and resistant *P. aeruginosa* isolates, resulting in substantially lower MIC values for both the compounds and LEV, as well as FICI indices ≤ 0.5. Additionally, the enhanced antibacterial efficacy of the combination therapy was confirmed by FE-SEM analysis, which revealed bacterial membrane disruption and severe cellular damage. The mechanistic rationale for this synergistic interaction may be related to an increase in bacterial cell wall permeability, thereby potentiating levofloxacin’s antibacterial activity and improving overall treatment outcomes. Taken together, the results indicate that triazoloquinoxaline derivatives can act as adjuvant candidates for levofloxacin-based therapy, enabling dose reduction of levofloxacin while potentially minimizing side effects and overcoming antimicrobial resistance. This work establishes a promising framework for further molecular investigations and in vivo studies aimed at developing novel synergistic antimicrobial strategies.

## Experimental

### Materials and instrumentations

All reagents were purchased from Merck and Sigma-Aldrich Company and used without further purification. Solvents were obtained from Merck (Darmstadt, Germany) and Pars-Chemie (Iran). Mueller Hinton Agar (MHA) and Mueller Hinton broth (MHB) were obtained from HiMedia Company (India). Dimethyl sulfoxide (DMSO) was used to prepare stock solutions of the synthetic compounds. Melting points of the compounds were determined using a Barnstead Electrothermal 9300 apparatus. Their proton and carbon nuclear magnetic resonance (^1^H- and^ 13^C-NMR) spectra were obtained in DMSO-d_6_ on a Bruker DRX-400 and a Bruker FT-500 spectrometer, using tetramethylsilane as an internal reference. Mass spectra (MS) were recorded with an Agilent Technology (HP) mass spectrometer operating at an ionization potential of 70 eV. FT-IR spectra were recorded as KBr pellets on a Nicolet Fourier Transform Infrared Spectrometer (FT-IR). Thin-layer chromatography (TLC), pre-coated Merck silica gel 60 F254 plates was used to monitor the progress of the reactions. The cellular morphological changes of bacterial strains were observed using field emission scanning electron spectroscopy (FE-SEM, TESCAN, MIRA 3, Czech Republic). At first, the surfaces of the samples were coated with a thin layer of gold via an Emitech SC7620 sputter coater (Quorum Technologies Ltd., East Sussex, UK). Sensitive microbial strains of *S. aureus* (ATCC 29213), *S. epidermidis* (ATCC 12228), *E. coli* (ATCC 25922), *P. aeruginosa* (ATCC 27853), and *C. albicans* (ATCC 10231) were prepared from the Microbial Banks of Pasteur Institute of Iran. Antibiotic-resistant bacterial isolates were obtained from hospitalized patients^53,54^. L929 (NCBI C161) cells was prepared from the Pasteur Institute of Iran Cell Bank.

## Chemistry

### General synthetic route of [1,2,4]-triazolo[4,3-α]quinoxaline derivatives

Figure 2 depicts the synthetic routes employed in this study.

### Synthesis of quinoxaline-2,3(1*H*,4*H*)-dione (1)

A mixture comprising benzene-1,2-diamine (92 mmol, 10 g), oxalic acid (139 mmol, 12.5 g), and 10% hydrochloric acid (30 mL) in 30 mL of water was stirred and heated at 100 °C for 2 h. Afterward, the reaction mixture was allowed to cool, filtered and washed with ethylacetate. A white powder was obtained in 95% yield. Melting point: 300 °C (literature: ≤ 300 °C)^38^.

### Synthesis of 2,3-dichloroquinoxaline (2)

Quinoxaline-2,3(1*H*,4*H*)-dione (12 mmol, 1.94 g) was reacted with thionyl chloride (10 mL) in the presence of a catalytic amount of DMF and refluxed at 70 °C for 3 h. Upon completion, the reaction mixture was cooled to room temperature, and excess SOCl_2_ was evaporated, and the solid dried in air. The resulting product, 2,3-dichloroquinoxaline, was obtained as a white crystalline solid with a yield of 95%. Melting point: 148–151 °C (literature: 148–150 °C)^39^. FT-IR (KBr, cm^− 1^): 3177.10 (CH aromatic), 1600.04 & 1490.03 (C = C aromatic), 1373.22 (C = N), 750.52 (C-Cl). MS (m/z): 199.1 (M^+^).

### Synthesis of 2-chloro-3-hydrazineylquinoxaline (3)

In the next step, 2,3-dichloroquinoxaline (**2**) (1 mmol, 0.197 g) was dissolved in ethanol (5 mL). Hydrazine hydrate (1.5 mmol, 0.48 mL) was then added, and the reaction mixture was stirred at room temperature overnight. The resulting solid precipitate was filtrated, washed with ethanol, and air-dried. A white powder was obtained in 90% yield. Melting point: 182–184 °C (Literature: 181–182 °C)^40^. FT-IR (KBr, cm-1): 3360.06 (NH_2_), 3350.26 (NH stretching), 3007.10 (CH aromatic), 1600.04 & 1490 (C = C aromatic), 1582.32 (NH bending), 749.90 (C-Cl). MS (m/z): 194.1 (M^+^).

### Synthesis of 4-chloro-[1,2,4]triazolo[4,3-a]quinoxaline and 4-chloro-1-methyl-[1,2,4]triazolo[4,3-a]quinoxaline (4a-b)

A mixture of 2-chloro-3-hydrazinoquinoxaline (3) (5 mmol, 1 g) and either triethyl orthoformate or of triethyl orthoacetate (5 mL) was refluxed for 10 min. After cooling to room temperature, the precipitate was washed with diethyl ether and air-dried to obtain compounds 4a-b as yellow solids in ≥85% yield^41^. Compound 4a: Yield: 97%. 1H-NMR (500 MHz, DMSO-d6) δ (ppm): 7.70 (1H, t, J = 8.06 Hz, Ar), 7.85 (1H, t, J = 8.06 Hz, Ar), 8.05 (1H, d, J = 8.06 Hz, Ar) 8.45 (1H, d, J = 8.06 Hz, Ar), 10.03 (1H, s, CH triazole). Compound 4b: Yield: 95%. 1H-NMR (500 MHz, DMSO-d6) δ ppm: 7.75 (1H, t, J = 5 Hz, Ar), 7.83 (1H, t, J = 5 Hz, Ar), 8.05 (1H, d, J = 5 Hz, Ar) 8.45 (1H, d, J = 5 Hz, Ar), 3.12 (3H, s, -CH3 triazole).

### Synthesis of final derivatives 5a-i

To synthesize the final derivatives, 2 mmol of either compounds **4a** or **4b** was reacted with 2.3 mmol of various aliphatic/aromatic amines in 15 mL of ethanol under reflux conditions for 4–5 h. After completion of the reaction, the solvent was evaporated under reduced pressure, and the solid residue was filtered, dried, and recrystallized from absolute ethanol to afford the corresponding target compounds.

*N-ethyl-[1*,*2*,*4]triazolo[4*,*3-α]quinoxalin-4-amine (****5a****)*: Yield: 79%. Faint yellow, m.p.: 149–151 °C. ^1^H-NMR (400 MHz, DMSO-d6) δ (ppm): 1.26 (3 H, d, *J* = 8 Hz, -CH_3_), 3.60 (2 H, m, -CH_2_-), 7.33 (1H, t, *J* = 8 Hz, Ar), 7.46 (1H, t, *J* = 8 Hz, Ar), 7.60 (1H, d, *J* = 8 Hz, Ar), 8.29 (1H, d, *J* = 8 Hz, Ar), 8.15 (1H, t, *J* = 8 Hz, -NH-), 10.24 (1H, s, CH triazole). ^13^C-NMR (101 MHz, DMSO-d6) δ (ppm): 14.86, 35.31, 116.56, 122.25, 123.48, 126.48, 127.97, 137.65, 138.36, 138.78, 145.92. FT-IR (KBr, cm^− 1^): 3303.5 (N-), 3024.9 (CH aromatic), 2885.6 (CH aliphatic), 1606.8 & 1467.5 (C = C aromatic), 1587.2 (C = N triazole). MS (m/z): 213.1 (M^+^).

*2-([1*,*2*,*4]triazolo[4*,*3-*α*]quinoxalin-4-ylamino)ethan-1-ol (****5b****)*: Yield: 84%. Faint yellow, m.p.: 237–239 °C (Lit. 239–241 °C). ^1^H-NMR (400 MHz, DMSO-d6) δ (ppm): 3.47(4H, t, *J* = 8 Hz, -CH_2_-), 6.15 (1H, s, -OH), 7.33 (1H, t, *J* = 8 Hz, Ar), 7.45 (1H, t, *J* = 8 Hz, Ar), 7.58 (1H, d, *J* = 8 Hz, Ar), 8.08 (1H, d, *J* = 8 Hz, Ar), 8.16 (1H, s, -NH-), 9.98 (1H, s, CH triazole). ^13^C-NMR (101 MHz, DMSO-d6) δ (ppm): 46.89, 47.99, 115.62, 122.68, 123.34, 126.11, 126.88, 137.66, 139.48, 145.46, 148.23. FT-IR (KBr, cm^− 1^): 3442.8 (OH), 3303.5 (NH), 3164.2 (CH aromatic), 2905.6 (CH aliphatic), 1676.5 & 1467.8 (C = C aromatic), 1537.2 (C = N triazole). MS (m/z): 229.2 (M^+^).

*N-propyl-[1*,*2*,*4]triazolo[4*,*3-α]quinoxalin-4-amine (****5c****)*: Yield: 79%. Faint yellow, m.p.: 172–174 °C (Lit. 173–175 °C). ^1^H-NMR (400 MHz, DMSO-d6) δ (ppm): 0.99 (3H, t, *J* = 8 Hz, -CH_3_), 1.75 (2H, m, -CH_2_-), 2.70 (2H, t, *J* = 8 Hz, -CH_2_-), 7.40 (1H, t, *J* = 8 Hz, Ar), 7.50 (1H, t, *J* = 8 Hz, Ar), 7.80 (1H, s, Ar), 8.22 (1H, d, *J* = 8 Hz, Ar), 7.80 (1H, s, -NH-), 10.07 (1H, s, CH triazole), ^13^C-NMR (101 MHz, DMSO-d6) δ (ppm): 13.36, 28.90, 38.35, 116.24, 116.77, 120.36, 127.90, 128.09, 128.88, 130.22,138.44, 145.11. FT-IR (KBr, cm^− 1^): 3328.4 (NH), 3059.7 (CH aromatic), 2925.4 (CH aliphatic), 1656.6 & 1455.1 (C = C aromatic), 1589.4 (C = N triazole). MS (m/z): 227.1 (M^+^).

*N-isobutyl-[1*,*2*,*4]triazolo[4*,*3-α]quinoxalin-4-amine (****5d****)*: Yield: 80%. Faint yellow, m.p.: 145–147 °C. ^1^H-NMR (400 MHz, DMSO-d6) δ (ppm): 0.96 (6H, d, *J* = 8 Hz, -CH_3_ & -CH_3_), 2.12 (2H, m, -CH-), 3.39 (2H, t, *J* = 8 Hz, -CH_2_-), 7.31 (1H, t, *J* = 8 Hz, Ar), 7.42 (1H, t, *J* = 8 Hz, Ar), 7.60 (1H, s, Ar), 8.30 (1H, d, *J* = 8 Hz, Ar), 8.15 (1H, d, *J* = 8 Hz, -NH-), 10.01 (1H, s, CH triazole). ^13^C-NMR (101 MHz, DMSO-d6) δ (ppm): 12.96, 14.52, 36.15, 124.79, 125.86, 127.88, 128.36, 130.63, 135.79, 138.34, 141.49, 148.59. FT-IR (KBr, cm^− 1^): 3328.4 (NH), 3194.00 (CH aromatic), 2925.4 (CH aliphatic), 1625.6 & 1455.1 (C = C aromatic), 1589.4 (C = N triazole). MS (m/z): 241.3 (M^+^).

*N-hexyl-[1*,*2*,*4]triazolo[4*,*3-α]quinoxalin-4-amine (****5e****)*: Yield: 85%. Faint yellow, m.p.: 163–165 °C. ^1^H-NMR (400 MHz, DMSO-d6) δ (ppm): 1.31 (3H, d, *J* = 4 Hz, -CH_3_), 1.46 (4H, m, -CH_2_- & -CH_2_-), 1.68 (2H, m, *J* = 4 Hz, -CH_2_-), 2.65 (2H, t, J = 8 Hz, -CH_2_-), 3.51 (2H, m, *J =* 4 Hz, -CH_2_-), 7.30 (1H, t, *J* = 4 Hz, Ar), 7.45 (1H, t, *J* = 8 Hz, Ar), 7.58 (1H, d, *J =* 4 Hz, Ar), 8.28(1H, d, *J* = 4 Hz, Ar), 8.16 (1H, t, *J* = 8 Hz, -NH-), 9.97 (1H, s, CH triazole). ^13^C-NMR (101 MHz, DMSO-d6) δ (ppm): 14.33, 22.56, 26.17, 29.00, 31.35, 116.58, 122.21, 123.39, 126.43, 127.91, 137.66, 138.40, 138.74, 146.05. FT-IR (KBr, cm^− 1^): 3328.4 (NH), 3059.7 (CH aromatic), 2925.4 (CH aliphatic), 1589.4 & 1455.1 (C = C aromatic), 1522.2 (C = N triazole). MS (m/z): 269.1 (M^+^).

*N-(4-methylbenzyl)-[1*,*2*,*4]triazolo[4*,*3-α]quinoxalin-4-amine (****5f****)*: Yield: 75%. Faint yellow, m.p.: 152–154 °C. ^1^H-NMR (400 MHz, DMSO-d6) δ (ppm): 2.25 (3H, s, -CH_3_), 4.72 (2H, d, *J* = 8 Hz, -CH_2_-), 7.11 (3H, t, *J* = 4 Hz, Ar), 7.33 (3H, t, *J* = 4 Hz, Ar), 7.44 (1H, d, *J* = 4 Hz, Ar), 7.59 (1H, d, *J* = 4 Hz, Ar), 8.16 ( 1H, d, *J* = 4 Hz, Ar), 8.16 (1H, t, *J* = 8 Hz, -NH-), 9.98 (1H, s, CH triazole). ^13^C-NMR (101 MHz, DMSO-d6) δ (ppm): 21.14, 43.34, 116.57, 122.42, 123.71, 126.59, 127.96, 127.98, 129.19, 136.18, 136.94, 137.42, 138.42, 138.77, 145.91. FT-IR (KBr, cm^− 1^): 3303.5 (NH), 3024.9 (CH aromatic), 2886.5 (CH aliphatic), 1606.5 & 1467.8 (C = C aromatic), 1537.2 (C = N triazole). MS (m/z): 289.2 (M^+^).

*N’-([1*,*2*,*4]triazolo[4*,*3-α]quinoxalin-4-yl)-2-phenylacetohydrazide (****5 g****)*: Yield: 75%. Faint yellow, m.p.: 265–266 °C. ^1^H-NMR (400 MHz, DMSO-d6) δ (ppm): 3.63 (2H, s, -CH_2_-), 7.21 (5H, m, *J* = 4 Hz, Ar), 7.74 (1H, t, *J* = 8 Hz, Ar), 7.84 (1H, t, *J* = 8 Hz, Ar), 8.45 (1H, d, *J* = 8 Hz, Ar), 8.07 (1H, d, *J* = 8 Hz, Ar), 8.24 (1H, s, -NH-), 9.84 (1H, s, -NH-), 10.25 (1H, s, CH triazole). ^13^C-NMR (101 MHz, DMSO-d6) δ (ppm): 40.57, 116.83, 126.94, 127.12, 128.67, 128.76, 129.44, 129.48, 129.53, 129.69, 130.80, 135.79, 136.19, 169.44, 170.06. FT-IR (KBr, cm^− 1^): 3328.4 (NH), 3194.00 (CH aromatic), 2925.4 (CH aliphatic), 1656.6 & 1455.1 (C = C aromatic), 1589.4 (C = N triazole).

*2-((1-methyl-[1*,*2*,*4]triazolo[4*,*3-α]quinoxalin-4-yl)amino)ethan-1-ol (****5 h****)*: Yield: 82%. Faint yellow, m.p.: 238–240 °C. ^1^H-NMR (400 MHz, DMSO-d6) δ (ppm): 3.65 (4H, t, *J* = 8 Hz, -CH_2_-), 4.87 (1H, s, -OH), 7.32 (1H, t, *J* = 8 Hz, Ar), 7.46 (1H, t, *J* = 8 Hz, Ar), 7.60 (1H, d, *J* = 8 Hz, Ar), 8.10 (1H, d, *J* = 8 Hz, Ar), 7.95 (1H, s, -NH-), 3.04 (3H, s, -CH_3_ triazole). ^13^C-NMR (101 MHz, DMSO-d6) δ (ppm): 18.28, 43.23, 44.98, 115.53, 122.56, 123.27, 126.05, 126.82, 137.69, 139.44, 145.34, 148.17. FT-IR (KBr, cm^− 1^): 3462.7 (OH), 3328.4 (NH), 3059.7 (CH aromatic), 2925.4 (CH aliphatic), 1656.6 & 1455.1 (C = C aromatic), 1522.2 (C = N triazole). MS (m/z): 243.2 (M^+^).

*N-butyl-1-methyl-[1*,*2*,*4]triazolo[4*,*3-α]quinoxalin-4-amine (****5i****)*: Yield: 84%. Yellow powder, m.p.: 150–153 °C. ^1^H-NMR (400 MHz, DMSO-d6) δ (ppm): 0.91 (3H, t, *J* = 8 Hz, -CH_3_), 1.39 (2H, m, -CH_2_-), 1.66 (2H, t, *J* = 8 Hz, -CH_2_-), 3.55 (2 H, t, *J* = 8 Hz, -CH_2_-), 7.29 (1H, t, *J* = 4 Hz, Ar), 7.43 (1H, t, *J* = 4 Hz, Ar), 8.13 (1H, d, *J*= 4 Hz, Ar), 7.57 (1H, *J* = 4 Hz, Ar), 8.08 (1H, d, *J*= 4 Hz, -NH-), 3.16 (3H, s, -CH_3_ triazole). ^13^C-NMR (101 MHz, DMSO-d6) δ (ppm): 14.26, 14.95, 20.17, 31.22, 116.22, 123.25, 123.90, 126.65, 127.46, 138.16, 140.04, 146.32, 148.77. FT-IR (KBr, cm^− 1^): 3340.8 (NH), 3077.1 (CH aromatic), 2813.4 (CH aliphatic), 1615.5 & 1483.7 (C = C aromatic), 1549.6 (C = N triazole). MS (m/z): 255.10 (M^+^).

*4-hydrazineyl-1-methyl-[1*,*2*,*4]triazolo[4*,*3-α]quinoxaline (****5j***): Yield: 73%. Faint yellow, m.p.: 262–264 °C. ^1^H-NMR (400 MHz, DMSO-d_6_) δ (ppm): 4.69 (2H, s, -NH_2_),7.32 (1H, t, *J* = 8 Hz, Ar), 7.71 (1H, t, *J* = 8 Hz, Ar), 7.66 (1H, d, *J*= 8 Hz, Ar), 8.10 (1H, d, *J* = 8 Hz, Ar), 9.45 (1H, s, -NH-), 3.04 (3H, s, -CH_3_ triazole). ^13^C-NMR (101 MHz, DMSO-d6) δ (ppm): 14.37, 115.53, 122.56, 123.27, 126.05, 126.82, 137.69, 139.44, 145.34, 148.17. FT-IR (KBr, cm^− 1^): 3442.8 (NH), 3303.5 (CH aromatic), 16.6.8 & 1467.5 (C = C aromatic), 1537.2 (C = N triazole).

### Experimental antimicrobial screening

The antimicrobial activity of [1,2,4]triazolo[4,3-a]quinoxaline derivatives was initially evaluated by the disc diffusion method as previously described^55^. The synthetic compounds (stock concentration of 1 mg/mL; test concentrations of 50 µg/mL in dichloromethane or ethyl acetate) were poured on each disc (6 mm diameter, Padtan Teb co.) and allowed to dry in air. The discs were then placed on MHA plates pre-inoculated with bacterial strains of *S. aureus* and *E. coli*, which were incubated at 37 °C for 24 h. After the incubation period, the DIZ were measured in millimeters, and compounds with DIZ ≥ 8 mm were selected for MIC tests (Data not shown).

The MIC and MBC of the compounds **5a-j** were determined according to a the Clinical and Laboratory Standards Institute (CLSI) guideline against bacterial strains of *S. aureus*, *S. epidermidis*, *E. coli*, *P. aeruginosa*, and fungal strain of *C. albicans*^56^. Eight serial dilutions were prepared from stock solutions (1 mg/mL in DMSO) in a concentration range from 250 to 1.95 µg/mL in 96-well microplates using MHB. Levofloxacin, penicillin, amikacin, and fluconazole were considered as positive controls, and DMSO was used as a negative control. A bacterial suspension in MHB was prepared from the culture plate after 24 h with approximately 5 × 10^5^ colony-forming units/mL (CFU/mL), and 100 µl of this suspension was inoculated into each well of a 96-well microplate. Subsequently, broth media (60 µL) and the test compounds/reference drugs were added to the first well to reach a final volume of 200 µL. A solution (100 µL) serially diluted from the first well was obtained by transferring 100 µL to the next well. This twofold dilution was continued down the plate, and 100 µL from the last well was removed. The plates were incubated at 37 °C for 16 h, and the turbidity in each well was observed. The well in which bacterial growth was completely inhibited was considered the MIC. Sterility and growth control wells were also included for each strain. The MIC well and two wells above the MIC value were transferred onto MHA plates by streaking and incubated at 37 °C for 24 h to determine the MBC and MFC of synthetic compounds. Each experiment was repeated at least three times.

### The evaluation of synergistic effects

The synergistic effect was assessed using a checkerboard assay in 96-well cell culture plates containing compounds **5b**, **5d**, and **5 h** and LEV in MHB^57^. Concentration gradients of LEV and the synthetic compounds were prepared in a 5 × 7 layout in the both horizontal and vertical directions, respectively. The first well served as the growth control. Bacterial suspensions were prepared in the same manner as described for the MIC test, with a higher concentration of 0.5 MacFarland for sensitive strains and 0.5 MacFarland for resistant isolates, and were then added to the plates. Controls included growth control (bacteria without compound), sterility control (medium only), and solvent control (DMSO). The plates were incubated at 37 °C for 16 h. The lowest concentration of the combination that completely inhibited bacterial growth was defined as the MIC for that specific combination. These values were used to calculate the fractional inhibitory concentration index (FICI) to evaluate the synergistic effect between the compounds and LEV, based on the following formula. Each experiment was repeated at least two times.

$$\:\mathrm{F}\mathrm{I}\mathrm{C}\mathrm{I}=\frac{\mathrm{M}\mathrm{I}\mathrm{C}\mathrm{a}\:\mathrm{i}\mathrm{n}\:\mathrm{c}\mathrm{o}\mathrm{m}\mathrm{b}\mathrm{i}\mathrm{n}\mathrm{a}\mathrm{t}\mathrm{i}\mathrm{o}\mathrm{n}}{\mathrm{M}\mathrm{I}\mathrm{C}\mathrm{a}}+\frac{\mathrm{M}\mathrm{I}\mathrm{C}\mathrm{a}{\prime\:}\:\mathrm{i}\mathrm{n}\:\mathrm{c}\mathrm{o}\mathrm{m}\mathrm{b}\mathrm{i}\mathrm{n}\mathrm{a}\mathrm{t}\mathrm{i}\mathrm{o}\mathrm{n}}{\mathrm{M}\mathrm{I}\mathrm{C}\mathrm{a}{\prime\:}}$$

MICa: MIC of synthetic compound; MICa’: MIC of LEV.

The FICI data was interpreted as follows: ≤0.5, synergistic; 0.5 < FICI < 1, partial synergistic; 1 < FICI < 4, additive or indifferent; and > 4, antagonistic effect, respectively^37^.

### Cell viability assay

The MTT assay protocol was used to evaluate the toxicity of compounds **5b**, **5d**, and **5 h** with suitable antimicrobial activity against L929 (NCBI C161) cells, following a previously described protocol^51^. The cells were cultured in Dulbecco’s Modified Eagle Medium (DMEM) supplemented with 10% Fetal Bovine Serum (FBS) in 96-well plates and then incubated at 37 °C in a 90% humidified atmosphere with 5% CO_2_. Subsequently, 1 × 10^4^ cells were seeded into each well along with culture medium (100 µL) and incubated at 37 °C to allow cell adhesion. After the incubation period, the supernatants were removed, and 90 µL of 8MIC, 4MIC, 2MIC, and MIC concentrations of each compound, along with FBS (10 µL), were added to the respective wells. The plates were then incubated again at 37 °C for 24 h. Following treatment, the culture medium was replaced with MTT solution (100 µL, 0.5 mg/mL), and the plates were incubated at 37 °C for 4 h. After the incubation period, the supernatant was discarded, and the purple formazan crystals were dissolved in isopropanol (100 µL). Finally, the plates were placed on a shaker for 15 min, and cell viability was measured at 570 nm using the following formula:

$$\:\mathrm{\%}\mathrm{V}\mathrm{i}\mathrm{a}\mathrm{b}\mathrm{i}\mathrm{l}\mathrm{i}\mathrm{t}\mathrm{y}=100-\left[(1-\frac{\mathrm{m}\mathrm{e}\mathrm{a}\mathrm{n}\:\mathrm{O}\mathrm{D}570\:\mathrm{o}\mathrm{f}\:\mathrm{S}\mathrm{a}\mathrm{m}\mathrm{p}\mathrm{l}\mathrm{e}\:}{\mathrm{m}\mathrm{e}\mathrm{a}\mathrm{n}\:\mathrm{O}\mathrm{D}570\:\mathrm{o}\mathrm{f}\:\mathrm{C}\mathrm{o}\mathrm{n}\mathrm{t}\mathrm{r}\mathrm{o}\mathrm{l}})\times\:100\right]$$

The selectivity index (SI) was calculated using the following formula:

$${\mathrm{SI}} = {\text{log }}\left[ {{\mathrm{CC}}_{{{\mathrm{5}}0}} } \right]/\left[ {{\mathrm{MIC}}} \right].$$

Positive SI values indicate greater selectivity of the compounds toward microorganisms, while negative SI values demonstrate higher toxicity to L929 cells (NCBI C161)^51^. Based on the SI results against sensitive strains, compounds **5b**, **5d**, and **5h** showed promising potential as alternative antibacterial agents.

### Hemolytic assay

The hemolytic activity of antibacterial derivatives **5b**, **5d**, and **5h** on erythrocytes was evaluated in accordance with ethical guidelines, following a previously described protocol^51^. A 15% (v/v) suspension of defibrinated sheep blood, purchased from Bahar Afshan Company (Iran), was prepared in normal saline. Then, 160 µL of normal saline and 40 µL of the synthetic compounds which showed suitable antibacterial activity (stock solution: 1 mg /mL in DMSO) were added to the first row of 96-well microplates. The synthetic compounds were then diluted across the wells to achieve different concentrations. Finally, 100 µL of pure blood suspension was added to all wells. Sterile normal saline served as the negative control, while 1% Triton-X 100 solution was used as a positive control for 100% erythrocyte lysis. The plates were incubated for 1 h at 37 °C, followed by centrifugation at 4,000 rpm for 10 min. The supernatants were carefully transferred to a new 96-well plate, and the absorbance was measured at 514 nm using a microplate reader (STAT FAX 2100, the USA). Ultimately, the hemolysis percentages were calculated as follows:

$${{\% Hemolysis = ~}}\left( {\frac{{{\mathrm{mean~OD~of~compound}} - {\mathrm{mean~OD~of~negative~control}}}}{{{\mathrm{mean~OD~of~positive~control}} - {\mathrm{mean~OD~of~negative~control}}}}} \right)~ \times {\mathrm{~}}100$$

### Analysis of cellular morphology using FE-SEM

Morphological changes of *P. aeruginosa* (ATCC 27853), *S. aureus* (ATCC 25923), and *S. epidermidis* (ATCC 12228) susceptible strains after exposure to the synergistic concentrations of compounds **5b**, **5d**, and **5h** with LEV were evaluated using the FE-SEM following a previously described protocol^58^. Initially, the susceptible bacterial strains were cultured on MHA and incubated at 37 °C for 24 h. Afterward, the bacterial suspensions were incubated with the synergistic combination well of compounds **5b**, **5d**, and **5h** alongside LEV for 18 h. The bacterial cells were then harvested by centrifugation at 1500 rpm for 10 min, followed by multiple washes using phosphate-buffered saline (PBS, pH 7.4) to remove any remaining culture medium. After washing, bacterial cells were fixed with 3% glutaraldehyde in PBS (pH 7.2) for 3 h, and subsequently washed three times with 0.1 M PBS (pH 7.2), each wash lasting 30 min. Post-fixation was performed with 1% osmium tetroxide in PBS (pH 7.2) for 1 h, and the samples were again washed by three times for 10 min with PBS to remove excess osmium tetroxide. Finally, the samples were dehydrated by water/ethanol gradient solutions (30%, 50%, 70%, 90%, and 100%). Complete drying of the samples was performed by the hexamethyldisilane reagent, and dried samples were mounted on aluminum foils using carbon adhesive. Samples were coated with a thin layer of gold under vacuum, and their morphological changes were observed using a field-emission scanning microscope. Control cells were untreated bacterial strains in culture medium.

## Supplementary Information

Below is the link to the electronic supplementary material.

Supplementary Material 1

## Data Availability

All data generated or analyzed during this study are included in this published article.

## References

[1] Karnwal, A. et al. Addressing the global challenge of bacterial drug resistance: insights, strategies, and future directions. *Front. Microbiol.* **16**, 1517772 (2025). 40066274 10.3389/fmicb.2025.1517772 PMC11891257

[2] Bouzammit, R. et al. Synthesis, crystal structure, DFT calculations, in-vitro and in-silico studies of novel chromone-isoxazoline conjugates as antibacterial and anti-inflammatory agents. *Sci. Rep.* **15**, 31103 (2025). 40851012 10.1038/s41598-025-11182-9 PMC12375725

[3] Provenzani, R. et al. Multisubstituted pyrimidines effectively inhibit bacterial growth and biofilm formation of Staphylococcus aureus. *Sci. Rep.* **11**, 7931 (2021). 10.1038/s41598-021-86852-5 PMC8041844 33846401

[4] Kabir, E. & Uzzaman, M. A review on biological and medicinal impact of heterocyclic compounds. *Results Chem.* **4**, 100606 (2022).

[5] Hameed, P. S. et al. Nitrothiophene carboxamides, a novel narrow spectrum antibacterial series: mechanism of action and efficacy. *Sci. Rep.* **8**, 7263 (2018). 29740005 10.1038/s41598-018-25407-7 PMC5940854

[6] Hassan, S. A. & Abdullah, M. N. Synthesis of new imidazole derivatives bearing a morpholine moiety: molecular docking, DFT (FOMs, MEP, RDG, ELF, LOL) analysis, and evaluation of antimicrobial, anti-inflammatory (In Vivo/In Vitro), toxicity, and hemolytic activities. *J. Mol. Struct.* 143286 (2025).

[7] Hassan, S. A. & Abdullah, M. N. Synthesis of new 4-thiazolidinone bearing thiazole, assess anticancer, and antimicrobial activities: insights from DFT, and molecular mocking. *J. Mol. Struct.* 143181 (2025).

[8] El-Attar, M. A. Z. et al. Design and synthesis of some new 1, 2, 4-triazolo (4, 3a) Quinoxaline derivatives as potential antimicrobialagents. *Med. chem.* **5**, 489–495 (2015).

[9] Patinote, C., Cirnat, N., Bonnet, P. A. & Deleuze-Masquéfa, C. Fused Azolo-Quinoxalines: candidates for medicinal Chemistry. A review of their biological applications. *Curr. Med. Chem.* **28**, 712–749 (2021). 32026768 10.2174/0929867327666200206114936

[10] Chawla, G., Gupta, O. & Pradhan, T. A review on multipurpose potential of bioactive heterocycle Quinoxaline. *ChemistrySelect* **8**, e202301401 (2023).

[11] Tariq, S., Somakala, K., Amir, M. & Quinoxaline An insight into the recent Pharmacological advances. *Eur. J. Med. Chem.* **143**, 542–557 (2018). 29207337 10.1016/j.ejmech.2017.11.064

[12] El-Naggar, M. A. et al. One pot synthesis of two potent ag (I) complexes with Quinoxaline ligand, X-ray structure, Hirshfeld analysis, antimicrobial, and antitumor investigations. *Sci. Rep.* **12**, 20881 (2022). 36463246 10.1038/s41598-022-24030-x PMC9719528

[13] Sharma, A., Deep, A., Marwaha, M. G. & Marwaha, R. K. Quinoxaline: A chemical moiety with spectrum of interesting biological activities. *Mini Rev. Med. Chem.* **22**, 927–948 (2022). 34579634 10.2174/1389557521666210927123831

[14] Waseem, A. M. et al. An insight into the therapeutic impact of quinoxaline derivatives: Recent advances in biological activities (2020–2024). *Res. Chem.* 101989 (2024).

[15] Bala Aakash, V., Ramalakshmi, N., Bhuvaneswari, S., Sankari, E. & Arunkumar, S. Comprehensive review on versatile pharmacology of quinoxaline derivative. *Russ J. Bioorg. Chem.* **48**, 657–677 (2022).

[16] Mamedov, V. A. *Quinoxalines: Synthesis, Reactions, Mechanisms and Structure*. (Springer, 2016).

[17] Khudair, M. et al. Triazole, properties and its medical applications.

[18] Matin, M. M. et al. Triazoles and their derivatives: Chemistry, synthesis, and therapeutic applications. *Front. Mol. Biosci.* **9**, 864286 (2022). 35547394 10.3389/fmolb.2022.864286 PMC9081720

[19] Reddivari, C. K. R. et al. Design, synthesis, biological evaluation and molecular Docking studies of 1, 4-disubstituted 1, 2, 3-triazoles: PEG-400: H2O mediated click reaction of fluorescent organic probes under ultrasonic irradiation. *Polycycl. Aromat. Compd.* **42**, 3953–3974 (2022).

[20] Guan, Q. et al. Triazoles in medicinal chemistry: physicochemical properties, bioisosterism, and application. *J. Med. Chem.* **67**, 7788–7824 (2024). 38699796 10.1021/acs.jmedchem.4c00652

[21] Kumar, S., Khokra, S. L. & Yadav, A. Triazole analogues as potential Pharmacological agents: A brief review. *Fut. J. Pharm. Sci.* **7**, 106 (2021). 34056014 10.1186/s43094-021-00241-3 PMC8148872

[22] Hahm, H. S. et al. Global targeting of functional tyrosines using sulfur-triazole exchange chemistry. *Nat. Chem. Biol.* **16**, 150–159 (2020). 31768034 10.1038/s41589-019-0404-5 PMC6982592

[23] Jørgensen, L. N. et al. Decreasing Azole sensitivity of Z. tritici in Europe contributes to reduced and varying field efficacy. *J. Plant. Dis. Prot.* **128**, 287–301 (2021).

[24] Tian, G. et al. Recent advances in 1, 2, 3-and 1, 2, 4-triazole hybrids as antimicrobials and their SAR: A critical review. *Eur. J. Med. Chem.* **259**, 115603 (2023). 37478558 10.1016/j.ejmech.2023.115603

[25] Al-Marhabi, A. R., Abbas, H. A. S. & Ammar, Y. A. Synthesis, characterization and biological evaluation of some Quinoxaline derivatives: A promising and potent new class of antitumor and antimicrobial agents. *Molecules* **20**, 19805–19822 (2015). 26540036 10.3390/molecules201119655 PMC6332220

[26] El Faydy, M. et al. Synthesis, biological properties, and molecular Docking study of novel 1, 2, 3-triazole-8-quinolinol hybrids. *ACS Omega*. **9**, 25395–25409 (2024). 38882066 10.1021/acsomega.4c03906 PMC11170742

[27] El-Attar, Z., Elbayaa, M. A. & Shaaban, R. Y. Synthesis of pyrazolo-1, 2, 4-triazolo [4, 3-a] quinoxalines as antimicrobial agents with potential Inhibition of DHPS enzyme. *Future Med. Chem.* **10**, 2155–2175 (2018). 30088415 10.4155/fmc-2018-0082

[28] Basavegowda, N. & Baek, K. H. Combination strategies of different antimicrobials: an efficient and alternative tool for pathogen inactivation. *Biomedicines* **10**, 2219 (2022). 36140320 10.3390/biomedicines10092219 PMC9496525

[29] Liu, Y. et al. Drug repurposing for next-generation combination therapies against multidrug-resistant bacteria. *Theranostics* **11**, 4910 (2021). 33754035 10.7150/thno.56205 PMC7978324

[30] Rehman, M. U. & Long, S. Progress and challenges in the development of Triazole antimicrobials. *Future Med. Chem.* **16**, 2451–2453 (2024). 39560011 10.1080/17568919.2024.2423596 PMC11622766

[31] Tahghighi, A. & Azerang, P. Click chemistry beyond metal-catalyzed cycloaddition as a remarkable tool for green chemical synthesis of antifungal medications. *Chem. Biol. Drug Des.* **103**, e14555 (2024). 38862260 10.1111/cbdd.14555

[32] Kubera, D. et al. Synergistic effect of synthetic derivatives of 1, 3, 4-thiadiazole with amphotericin B in antifungal therapy. *Sci. Rep.* **15**, 16663 (2025). 40360640 10.1038/s41598-025-01075-2 PMC12075838

[33] Islam, M. I. et al. Antimicrobial activity of IDD-B40 against drug-resistant Mycobacterium tuberculosis. *Sci. Rep.* **11**, 740 (2021). 33436895 10.1038/s41598-020-80227-y PMC7804135

[34] González, A. et al. Identifying potential novel drugs against Helicobacter pylori by targeting the essential response regulator HsrA. *Sci. Rep.* 911294 (2019). 10.1038/s41598-019-47746-9 PMC6683298 31383920

[35] Fang, Z. et al. A quinoline-based FtsZ inhibitor for the study of antimicrobial activity and synergistic effects with β-lactam antibiotics. *J. Pharmacol. Sci.* **137** (3), 283–289 (2018). 30057277 10.1016/j.jphs.2018.07.005

[36] Rogers, S. A., Huigens, R. W. III, Cavanagh, J. & Melander, C. Synergistic effects between conventional antibiotics and 2-aminoimidazole-derived antibiofilm agents. *Antimicrob. Agents Chemother.* **54**, 2112–2118 (2010). 20211901 10.1128/AAC.01418-09 PMC2863642

[37] Srivastava, R. et al. Synthesis, antibacterial activity, synergistic effect, cytotoxicity, Docking and molecular dynamics of benzimidazole analogues. *Comput. Biol. Chem.* **76**, 1–16 (2018). 29857255 10.1016/j.compbiolchem.2018.05.021

[38] Abbass, E. M., Khalil, A. K., Mohamed, M. M., Eissa, I. H. & El-Naggar, A. M. Design, efficient synthesis, Docking studies, and anticancer evaluation of new quinoxalines as potential intercalative Topo II inhibitors and apoptosis inducers. *Bioorg. Chem.* **104**, 104255 (2020). 32927130 10.1016/j.bioorg.2020.104255

[39] Mwakwari, S. C., Bess, L. S., Bazin, H. G. & Johnson, D. A. Efficient tin-mediated synthesis of lysophospholipid conjugates of a TLR7/8-active imidazoquinoline. *Tetrahedron Lett.* **57**, 2093–2096 (2016). 32863447 10.1016/j.tetlet.2016.03.110 PMC7451898

[40] Al Ward, M. M. S., Abdallah, A. E., Zayed, M. F., Ayyad, R. R. & El-Zahabi, M. A. Design, synthesis and biological evaluation of newly triazolo-quinoxaline based potential Immunomodulatory anticancer molecules. *J. Mol. Struct.* **1298**, 137041 (2024).

[41] Bi, X. et al. Design, synthesis and biological evaluation of novel 4, 5-dihydro-[1, 2, 4] Triazolo [4, 3-f] pteridine derivatives as potential BRD4 inhibitors. *Bioorg. Med. Chem.* **27** (13), 2813–2821 (2019). 31079968 10.1016/j.bmc.2019.05.006

[42] Bush, L. M. & Williams, J. Leuconostoc lactis bacteremia and neutropenic fever: an infrequently encountered Vancomycin-Resistant Gram-Positive cocci: A case report and review. *Infect. Dis. Clin. Pract.* **31**, e1234 (2023).

[43] Burke, Ó., Zeden, M. S. & O’Gara, J. P. The pathogenicity and virulence of the opportunistic pathogen *Staphylococcus epidermidis*. *Burke, Órla, Merve S Zeden, James P O’Gara 2024 The Pathog Virulence Opportunistic Pathog Staphylococcus Epidermidis Virulence 15(1) 2359483Virulence*. **15**, 2359483 (2024). 10.1080/21505594.2024.2359483 PMC11178275 38868991

[44] Letizia, M., Diggle, S. P. & Whiteley, M. Pseudomonas aeruginosa: ecology, evolution, pathogenesis and antimicrobial susceptibility. *Nat Rev. Microbiol.* 1–17 (2024). 10.1038/s41579-025-01193-8 PMC13064840 40442328

[45] Talapko, J. et al. Candida albicans—the virulence factors and clinical manifestations of infection. *J. Fungi*. **7**, 79 (2021). 10.3390/jof7020079 PMC7912069 33499276

[46] Henen, M. A., El Bialy, S. A. A., Goda, F. E., Nasr, M. N. A. & Eisa, H. M. [1,2,4]Triazolo[4,3-a]quinoxaline: Synthesis, antiviral, and antimicrobial activities. *Med. Chem. Res.* **21**, 2368–2378 (2012).

[47] Ayoup, M. S. et al. Synthesis, Docking, and evaluation of. *J. Heterocycl. Chem.* **53** (2016).

[48] Tambe, V., Ditani, A., Rajpoot, K. & Tekade, R. K. Pharmacokinetics aspects of structural modifications in drug design and therapy. In: *Biopharmaceutics and Pharmacokinetics Considerations*. 83–108 (Elsevier, 2021).

[49] Fesatidou, M., Petrou, A. & Athina, G. Heterocycle compounds with antimicrobial activity. *Curr. Pharm. Des.* **26**, 867–904 (2020). 32026773 10.2174/1381612826666200206093815

[50] Hassan, S. A., Ziwar, J. B., muhammed Aziz, D. & Abdullah, M. N. Sonochemical synthesis of new Thiazolidin-4-one derivatives as potent anticancer and antimicrobial agents with Docking design, and energy gap Estimation. *J. Mol. Struct.* **1301**, 137282 (2024).

[51] Ghods, M., Almasirad, A. & Tahghighi, A. Synthesis and in vitro anti-bacterial activity of novel quinoline-based aryl/heteroaryl amide hybrids. *J. Mol. Struct.* **1335**, 141923 (2025).

[52] Xiao, G., Li, J. & Sun, Z. The combination of antibiotic and non-antibiotic compounds improves antibiotic efficacy against multidrug-resistant bacteria. *Int. J. Mol. Sci.* **24**, 15493 (2023). 37895172 10.3390/ijms242015493 PMC10607837

[53] Pahlevani, M. et al. Antibacterial and wound healing effects of PEG-coated ciprofloxacin-loaded ZIF-8 nanozymes against ciprofloxacin-resistant Pseudomonas aeruginosa taken from burn wounds. *Front. Pharmacol.* **16**, 1556335 (2025). 41020001 10.3389/fphar.2025.1556335 PMC12461262

[54] Zafari, M. et al. Effects of cefazolin-containing niosome nanoparticles against methicillin-resistant Staphylococcus aureus biofilm formed on chronic wounds. *Biomed. Mater.* **16**, 35001 (2021). 10.1088/1748-605X/abc7f2 33650546

[55] Nabizadeh, M., Naimi-Jamal, M. R., Rohani, M., Azerang, P. & Tahghighi, A. Hydrazone analogues with promising antibacterial profiles: Synthesis, morphology, in vitro and in Silico approaches. *Lett. Appl. Microbiol.* **75**, 667–679 (2022). 35334115 10.1111/lam.13692

[56] Clinical and Laboratory Standards Institute (Ed.). *Methods for Dilution Antimicrobial Susceptibility Tests for Bacteria that Grow Aerobically: M07–A10; Approved Standard* 10th edn (Clinical Laboratory Standards, 2015).

[57] Namivandi-Zangeneh, R. et al. Synergy between synthetic antimicrobial polymer and antibiotics: a promising platform to combat multidrug-resistant bacteria. *ACS Infect. Dis.* **5**, 1357–1365 (2019). 30939869 10.1021/acsinfecdis.9b00049

[58] Saberi Harooni, N. et al. Antibacterial efficacy of pyranopyrimidinone derivatives synthesized using a facile one-pot reaction. *Res. Chem. Intermed.*.1–18 (2024).

