# Supplementary material for: Assessment of antimicrobial activity of [1,2,4]triazolo[4, 3-a]quinoxaline derivatives individually and in combination with levofloxacin
Source: Sci Rep. 2026 Feb 19;16:9902. doi: 10.1038/s41598-026-39141-y (PMC13018230; doi:10.1038/s41598-026-39141-y)

**N-propyl-[1,2,4]triazolo[4,3-a]quinoxalin-4-amine(5c):**

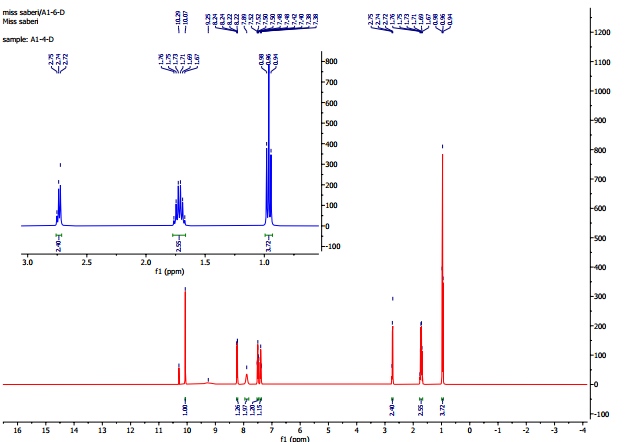


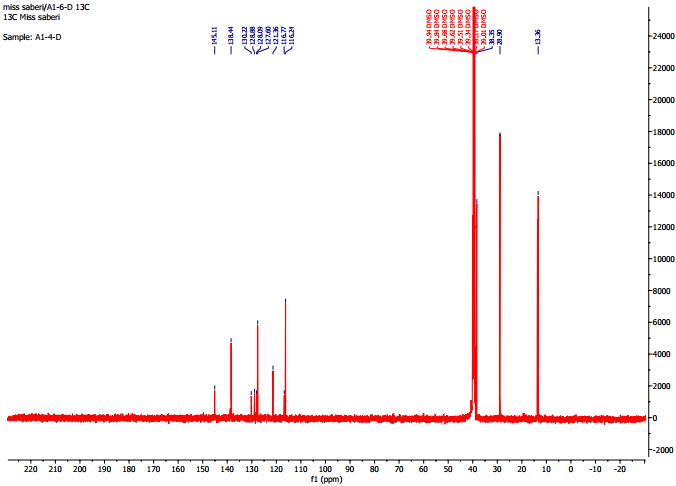


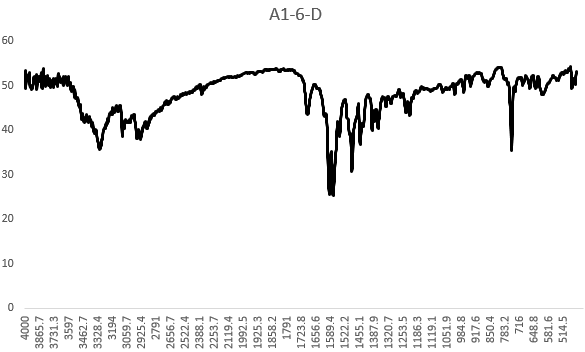


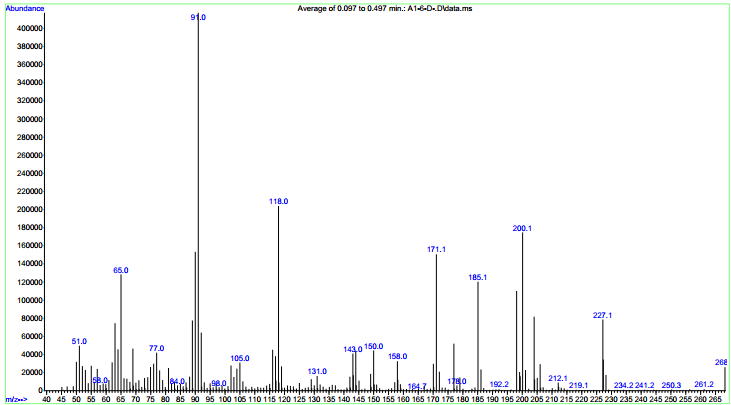


**N-isobutyl-[1,2,4]triazolo[4,3-a]quinoxalin-4-amine(5d):**

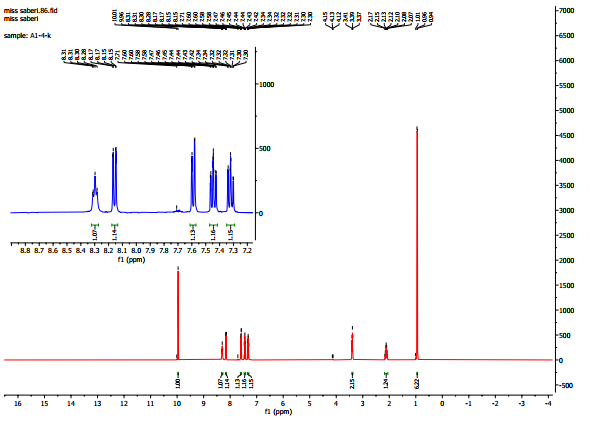


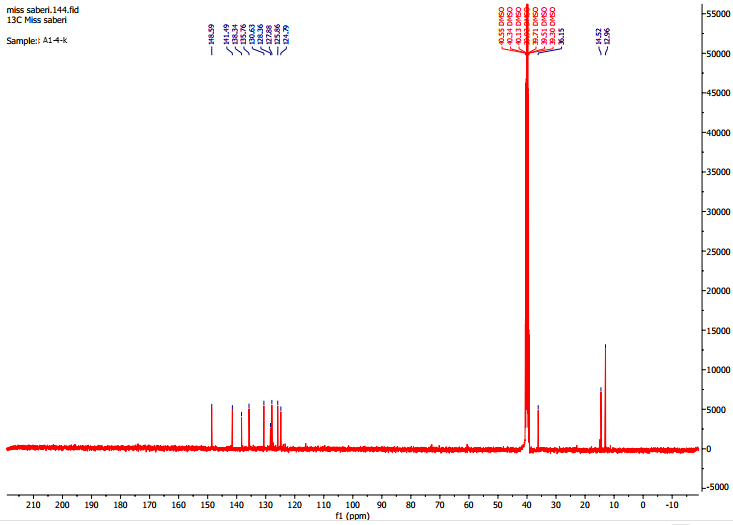


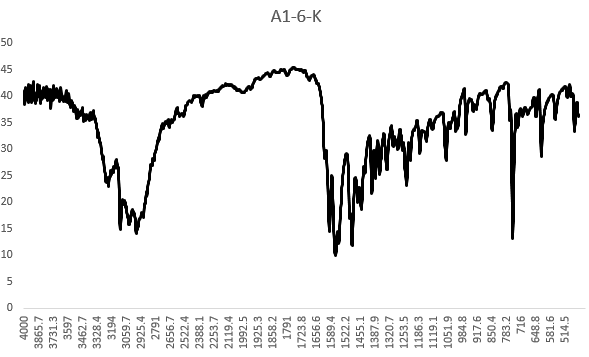


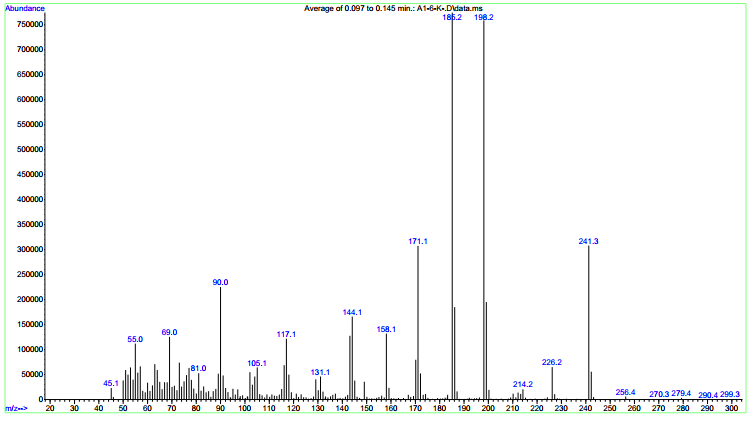


**N-hexyl-[1,2,4]triazolo[4,3-a]quinoxalin-4-amine (5e):**

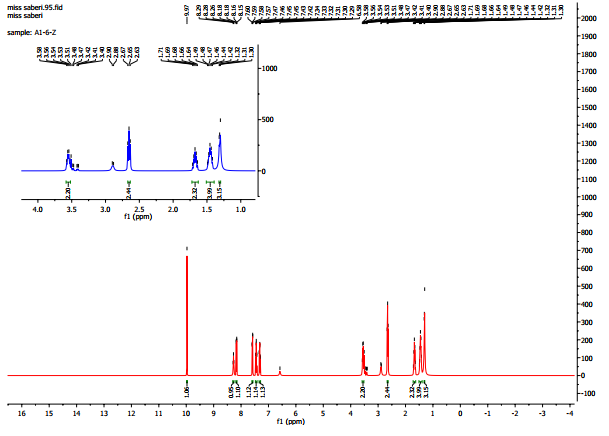


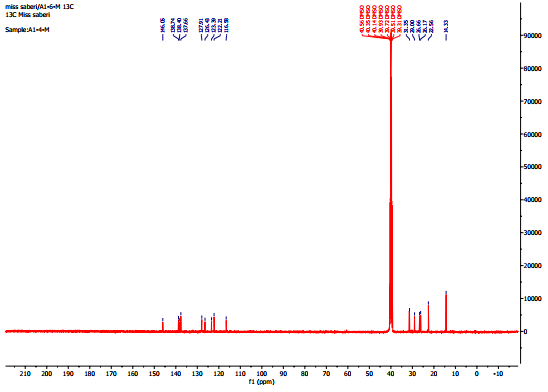


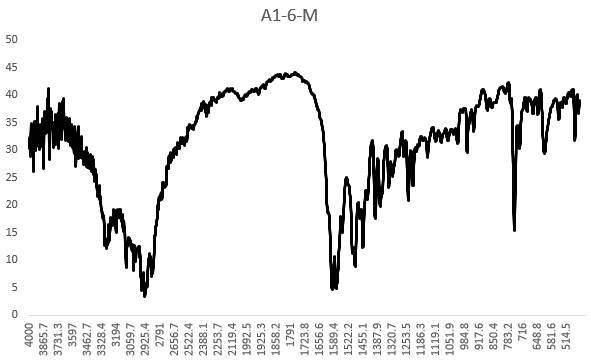


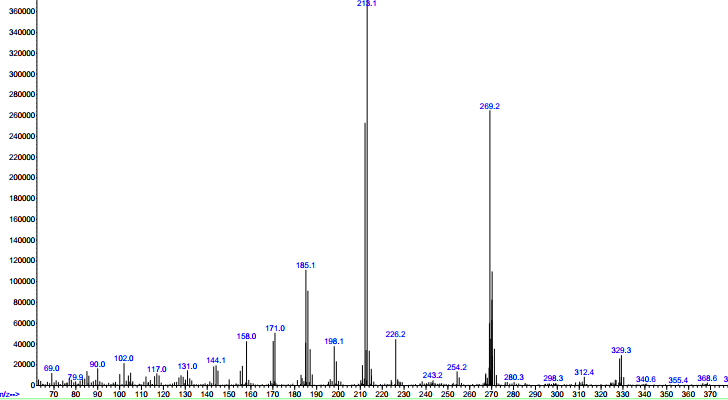


**N-ethyl-[1,2,4]triazolo[4,3-a]quinoxalin-4-amine (5a):**

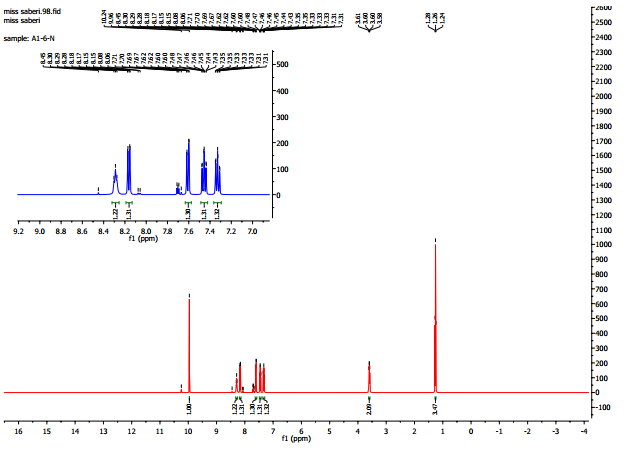


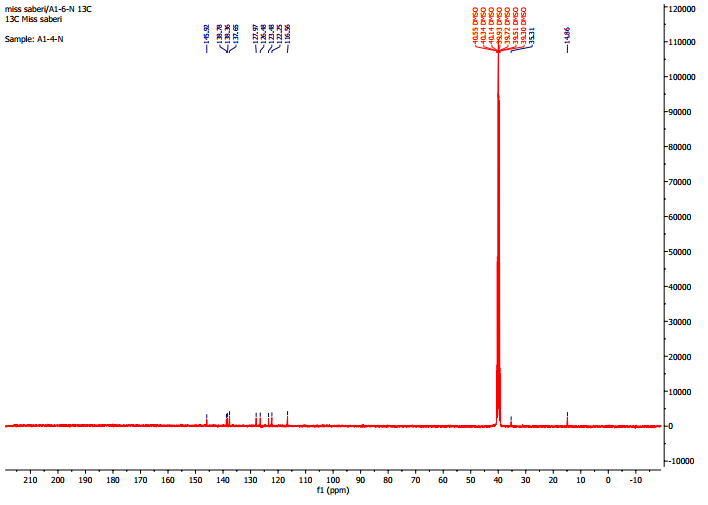


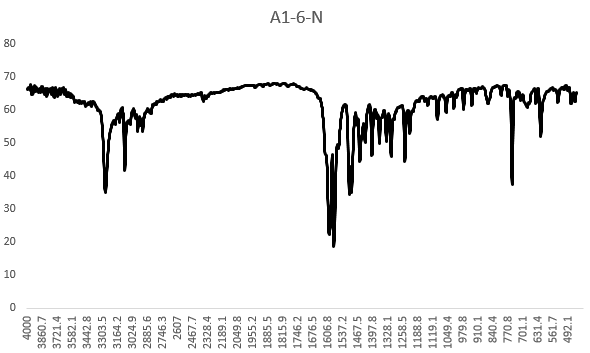


**
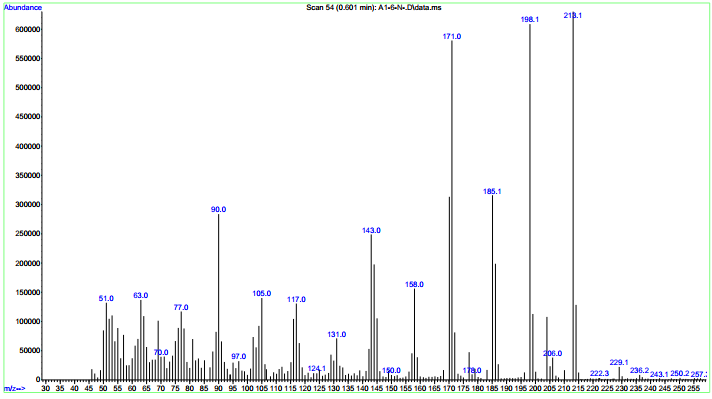
**

**2-([1,2,4]triazolo[4,3-a]quinoxalin-4-ylamino)ethan-1-ol(5b):**

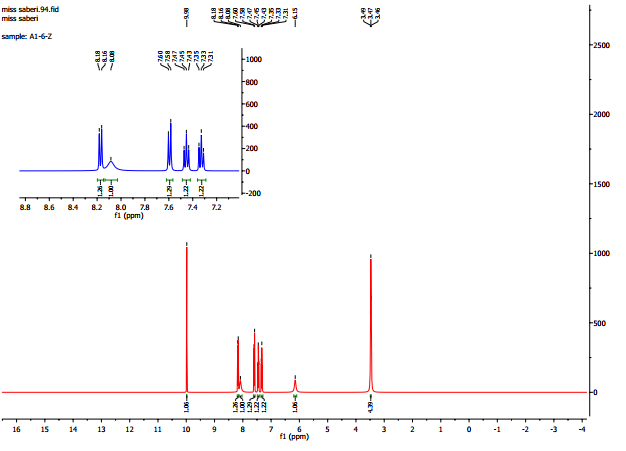


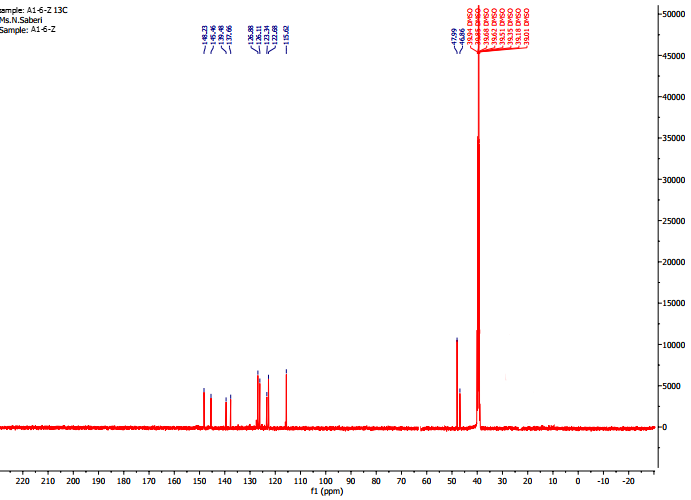


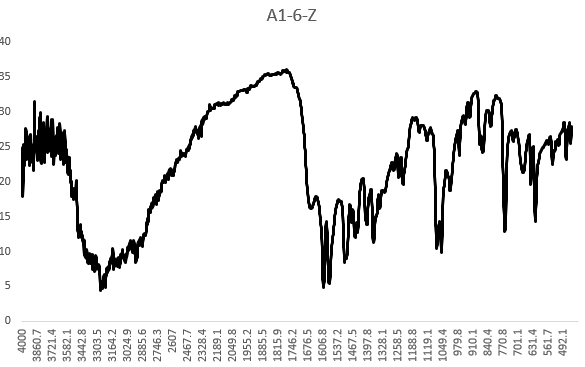


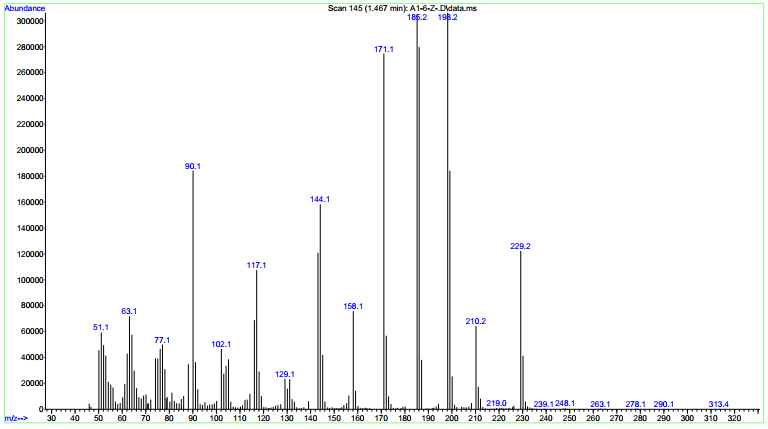


**N-(4-methylbenzyl)-[1,2,4]triazolo[4,3-a]quinoxalin-4-amine (5f):**

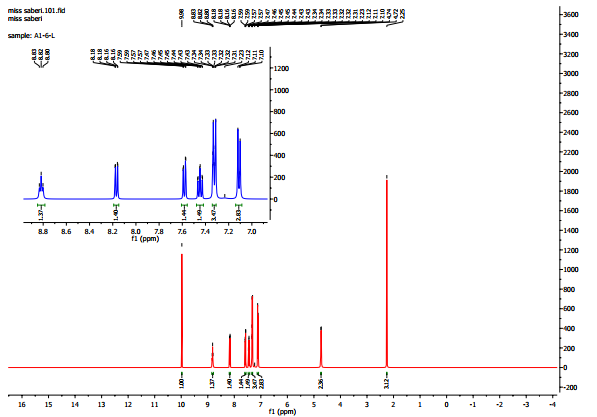


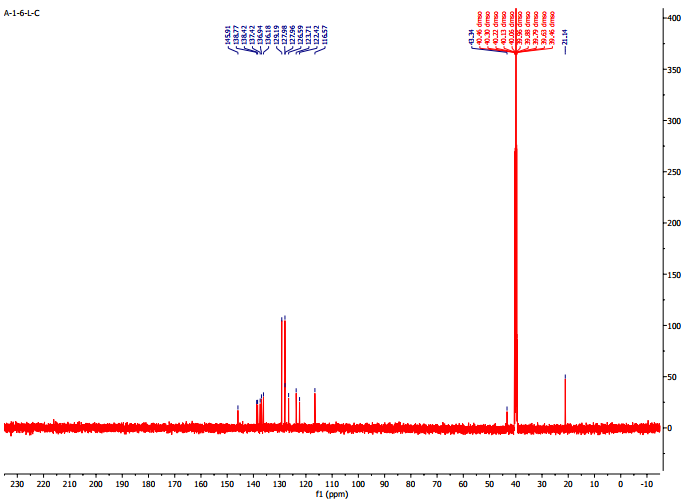


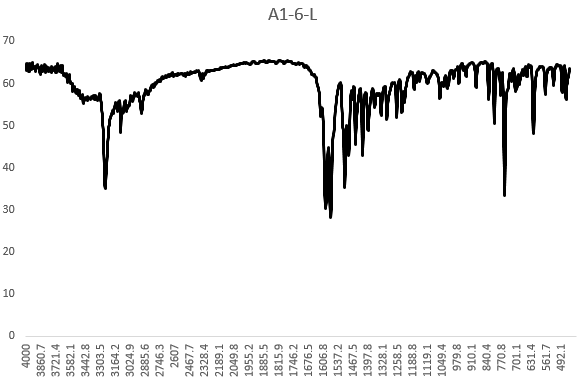


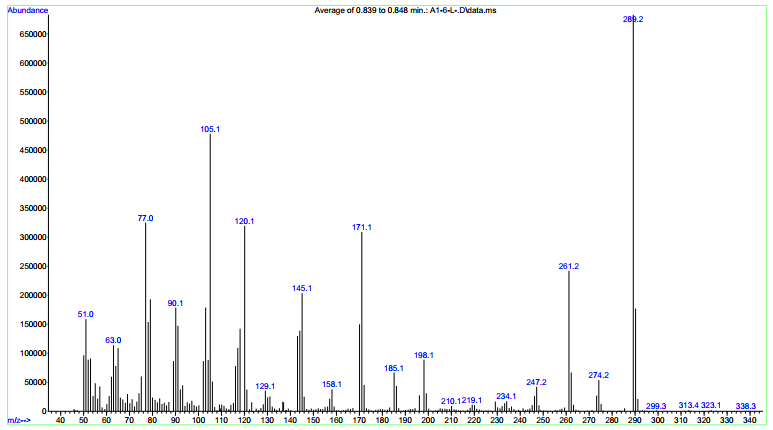


**N'-([1,2,4]triazolo[4,3-a]quinoxalin-4-yl)-2-phenylacetohydrazide (5g):**

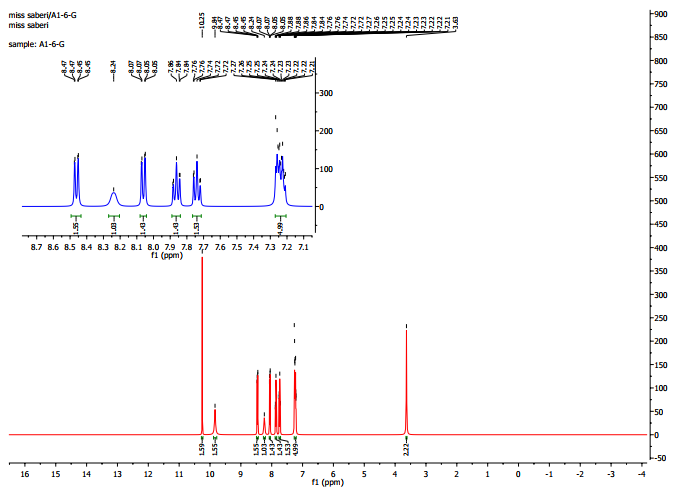


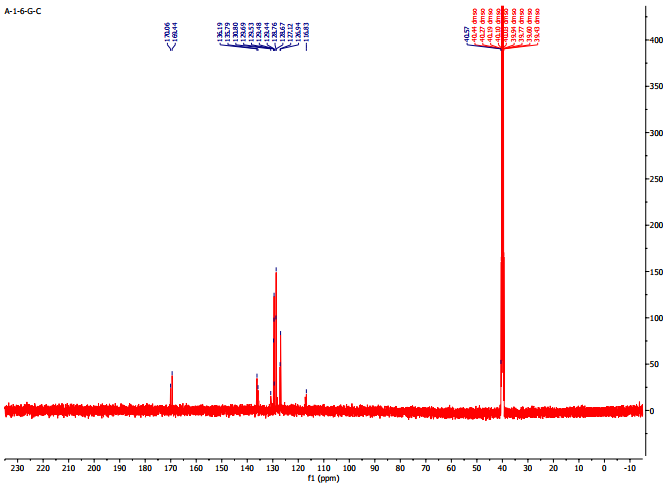


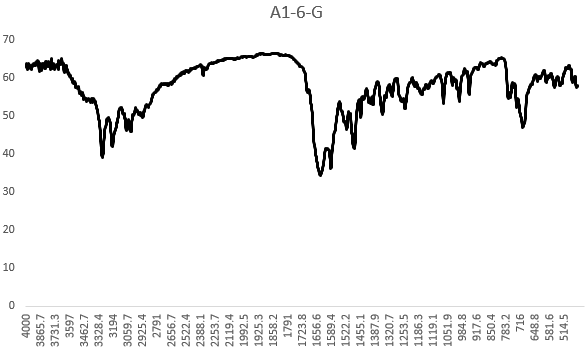


**2-((1-methyl-[1,2,4]triazolo[4,3-a]quinoxalin-4-yl)amino)ethan-1-ol (5h):**

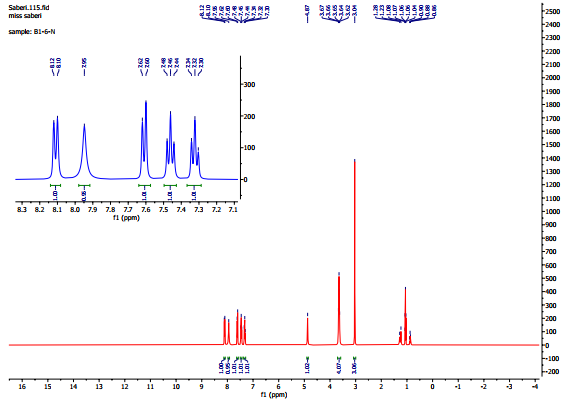


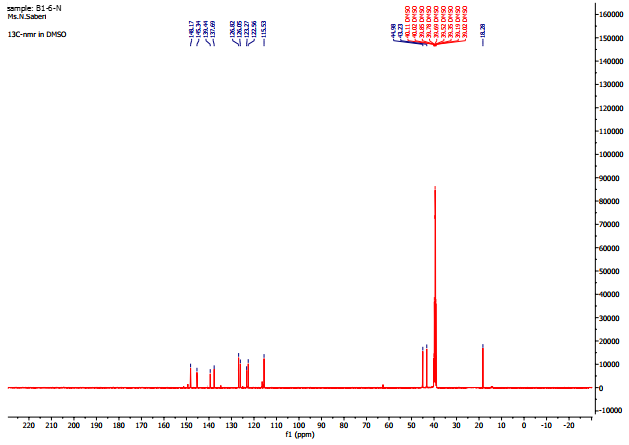


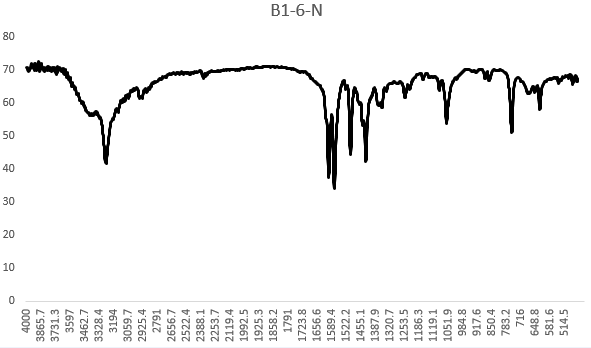


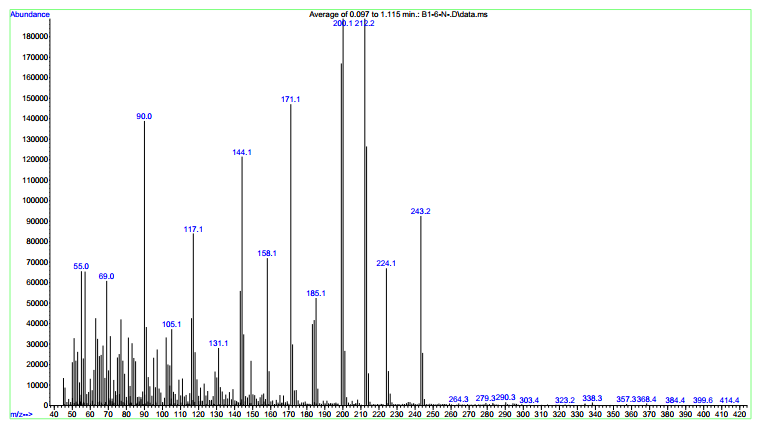


**N-butyl-1-Methyl-[1,2,4]triazolo[4,3-a]quinoxalin-4-amine(5i):**

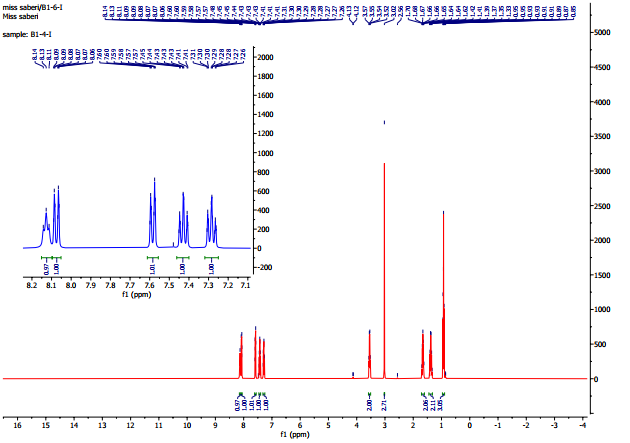


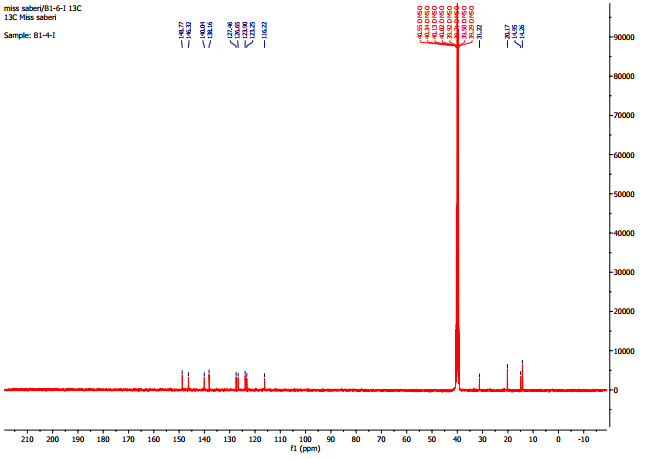


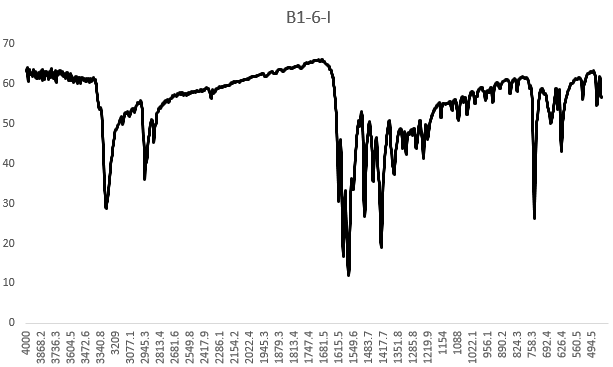


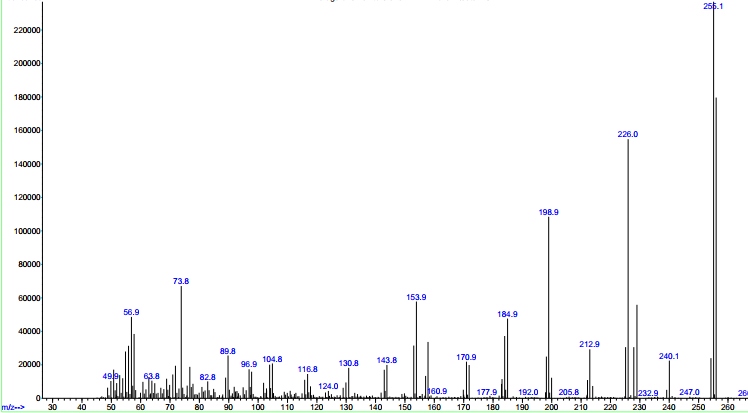


**4-hydrazineyl-[1,2,4]triazolo[4,3-a]quinoxaline (5j):**

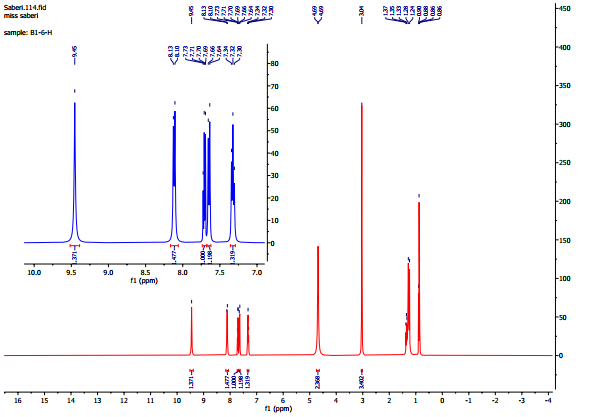


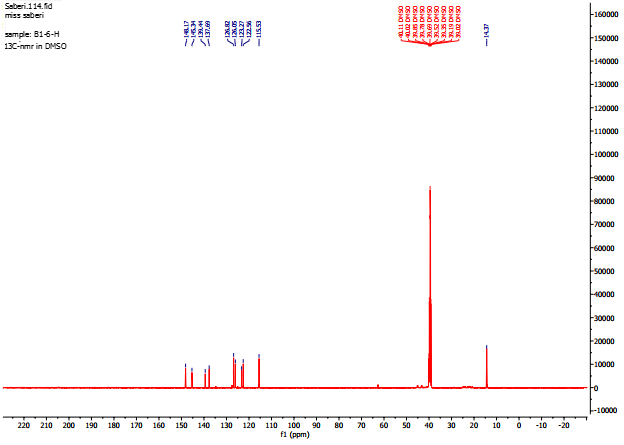


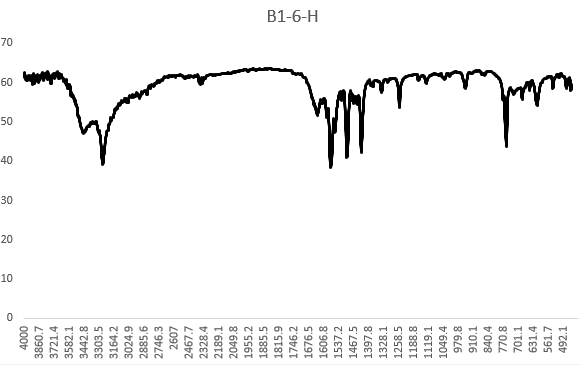

Supplement: Supplementary file 1 — Supplementary Material 1 [file 41598_2026_39141_MOESM1_ESM.docx]
